# The Lablite project: A cross-sectional mapping survey of decentralized HIV service provision in Malawi, Uganda and Zimbabwe

**DOI:** 10.1186/1472-6963-14-352

**Published:** 2014-08-19

**Authors:** Adrienne K Chan, Deborah Ford, Harriet Namata, Margaret Muzambi, Misheck J Nkhata, George Abongomera, Ivan Mambule, Annabelle South, Paul Revill, Caroline Grundy, Travor Mabugu, Levison Chiwaula, Fabian Cataldo, James Hakim, Janet Seeley, Cissy Kityo, Andrew Reid, Elly Katabira, Sumeet Sodhi, Charles F Gilks, Diana M Gibb, the Lablite Team

**Affiliations:** Dignitas International, Zomba, Malawi; Division of Infectious Diseases, Department of Medicine, University of Toronto, Toronto, Canada; MRC Clinical Trials Unit at University College London, London, U.K; Joint Clinical Research Centre, Kampala, Uganda; University of Zimbabwe, Harare, Zimbabwe; Infectious Diseases Institute, Makerere University, Mulago, Uganda; Centre for Health Economics, University of York, York, UK; MRC/UVRI Uganda Research Unit of AIDS, Entebbe, Uganda; Department of Family and Community Medicine, University of Toronto, Toronto, Canada; Department of Family and Community Medicine, University Health Network, Toronto Western Hospital, Toronto, Canada; School of Population Health, University of Queensland, Queensland, Australia

**Keywords:** HIV services, Sub-Saharan Africa, Antiretroviral therapy rollout, Primary care health facilities, Stock-outs, Decentralization

## Abstract

**Background:**

In sub-Saharan Africa antiretroviral therapy (ART) is being decentralized from tertiary/secondary care facilities to primary care. The Lablite project supports effective decentralization in 3 countries. It began with a cross-sectional survey to describe HIV and ART services.

**Methods:**

81 purposively sampled health facilities in Malawi, Uganda and Zimbabwe were surveyed.

**Results:**

The lowest level primary health centres comprised 16/20, 21/39 and 16/22 facilities included in Malawi, Uganda and Zimbabwe respectively. In Malawi and Uganda most primary health facilities had at least 1 medical assistant/clinical officer, with average 2.5 and 4 nurses/midwives for median catchment populations of 29,275 and 9,000 respectively. Primary health facilities in Zimbabwe were run by nurses/midwives, with average 6 for a median catchment population of 8,616. All primary health facilities provided HIV testing and counselling, 50/53 (94%) cotrimoxazole preventive therapy (CPT), 52/53 (98%) prevention of mother-to-child transmission of HIV (PMTCT) and 30/53 (57%) ART management (1/30 post ART-initiation follow-up only). All secondary and tertiary-level facilities provided HIV and ART services. In total, 58/81 had ART provision. Stock-outs during the 3 months prior to survey occurred across facility levels for HIV test-kits in 55%, 26% and 9% facilities in Malawi, Uganda and Zimbabwe respectively; for CPT in 58%, 32% and 9% and for PMTCT drugs in 26%, 10% and 0% of facilities (excluding facilities where patients were referred out for either drug). Across all countries, in facilities with ART stored on-site, adult ART stock-outs were reported in 3/44 (7%) facilities compared with 10/43 (23%) facility stock-outs of paediatric ART. Laboratory services at primary health facilities were limited: CD4 was used for ART initiation in 4/9, 5/6 and 13/14 in Malawi, Uganda and Zimbabwe respectively, but frequently only in selected patients. Routine viral load monitoring was not used; 6/58 (10%) facilities with ART provision accessed centralised viral loads for selected patients.

**Conclusions:**

Although coverage of HIV testing, PMTCT and cotrimoxazole prophylaxis was high in all countries, decentralization of ART services was variable and incomplete. Challenges of staffing and stock management were evident. Laboratory testing for toxicity and treatment effectiveness monitoring was not available in most primary level facilities.

**Electronic supplementary material:**

The online version of this article (doi:10.1186/1472-6963-14-352) contains supplementary material, which is available to authorized users.

## Background

Most HIV-infected individuals on antiretroviral therapy (ART) in low and middle-income countries are treated following the World Health Organization (WHO) public health approach [1]. The public sector provides one standard first-line regimen, with alternative drug substitutions for anti-tuberculosis (TB) co-therapy or first-line regimen toxicity [1]. When first-line failure occurs, the patient switches to a standard boosted-protease inhibitor (PI)-based second-line regimen [1]. National guidelines specify simple ART formularies with few combination drugs in order to facilitate procurement and supply-chain management [2–4]. Decentralization (delivery of services outside specialist centres) and task shifting (delegation of routine services to lower level relevant cadre) are key components of equitable public sector roll-out to ensure access to ART beyond ART facilities in tertiary centres and those operating in well-supported research programs [5–13]. Access to HIV testing and ART has been prioritized over laboratory services for monitoring ART toxicity and identifying treatment failure and the need to switch to second-line [14]. This has enabled large numbers of individuals to access ART and to remain on therapy [15]. It will remain the bedrock for further service expansion towards universal ART access, particularly in sub-Saharan African countries with generalized HIV epidemics, constrained health budgets and fragile health systems.

Funding for HIV and AIDS programs is generally static and has even recently declined in some resource constrained countries [16]. The “first-line/second-line” paradigm and the ensuing simplified formularies are not in question and decentralization remains a core policy objective in most countries. Despite robust clinical trials evidence demonstrating that ART may be given in both adults and children without routine laboratory monitoring [17–19], there remains an on-going debate about whether to prioritize limited funding towards laboratory capacity building for monitoring or expanding access to treatment [20, 21]. The 2013 WHO guidelines [22] promote both wider access to viral load monitoring and expanded entry into care (through raising the CD4 threshold for ART initiation and the ‘B plus’ approach to prevention of mother-to-child transmission whereby pregnant women start ART for life), without specific guidance on how to prioritize across these different aspirational approaches.

As these debates continue, many countries continue to decentralize their ART programs and policymakers face the challenge of prioritizing equity in access to ART with laboratory services and other health systems gaps. Although there are numerous studies describing decentralized ART programs in sub-Saharan African countries [5, 6, 9, 10, 23–27], few have described the state of national ART roll-out at the lowest level of decentralized primary service delivery. Moreover these studies [28, 29] have assessed epidemiologic program outcomes (mortality and lost to follow-up) but have generally not described HIV service decentralization through a health systems lens. There also remains a need to gather evidence on the safety, efficacy and cost-effectiveness of decentralization of services as clinically directed monitoring is operationalized in the context of limited laboratory support, outside of national centres of excellence.

Malawi, Uganda and Zimbabwe account for about 11% of people living with HIV globally and national ART coverage rates from 2012 UNAIDS data were 70%, 64% and 80% respectively, increased from 67%, 54% and 77% in 2011 [15]. Public sector scale-up of ART in these countries began about a decade ago, but decentralization and ART coverage at the lowest level of health services remain variable [30]. The Lablite project (http://www.lablite.org; 2011–2015) is a multi-country implementation project in these three countries to evaluate whether decentralized ART care can be delivered effectively at lower level health centres with limited laboratory services, and to assess the economic implications of decentralization employing a low technology, task-shifting strategy. Lablite began with a cross-sectional survey of representative health facilities to describe and compare national and inter-country delivery of training, clinical care and use of laboratories and monitoring in ART roll-out, providing a baseline for the project; this baseline survey is described here.

## Methods

### Study setting

Malawi (population 15 million), had a HIV prevalence of 11% among those aged 15–49 in 2012 [15]. ART coverage had expanded dramatically from 9 sites in 2003 to over 550 sites (67 private sector) at the time of initiation of the baseline study in October 2011, with over 275,000 HIV-positive individuals alive and on treatment [31]. The public health system in Malawi is organized into 3 levels: a) *primary:* consisting of community initiatives, health posts, dispensaries, maternity units, health centres (HC) which provide outpatient, antenatal, maternity, immunization and outreach services, and a transitional level of community and rural hospitals which provide inpatient care; b) *secondary:* district hospital referral centres that provide inpatient and outpatient services and are primarily based in towns; c) *tertiary:* 4 central hospitals that provide specialist referral health services for their respective health zones. A mid-level supervisory structure is organized geographically into 5 zonal health support offices, which provides support to a cluster of district health management teams who are responsible for coordinating the secondary and primary health facilities as well as the referral system within each district.

Uganda (population 34.5 million) had a prevalence of 7% among those aged 15–49 in 2012 [15]. It has the most mature ART program of the three countries, with availability of medication since 1998 through NGOs, the private sector and academic institutions. At the time of the baseline survey, end 2011, 313,000 HIV positive-individuals were on ART [32]. Provision was as per the 2009 national ART guidelines [3]. Health facilities are categorized according to the area served and services provided as: a) Health Centre II (HC II) which serves a parish with population ~5,000 and provides outpatient, antenatal, immunization and outreach services; b) Health Centre III (HC III) which serves a sub-county and additionally provides inpatient care and environmental health; c) Health Centre IV (HC IV) which serves a sub-district and additionally provides surgery, supervision of the lower HCs, data collection and health service planning. There are also 57 government hospitals, including 10 regional referral and teaching hospitals. For the purposes of comparison across countries HC IIs and national hospitals were not included and health facilities were classified as follows: a) *primary*: HC IIIs; b) *secondary*: HC IVs and district hospitals, as their roles in ART provision are similar; and c) *tertiary*: regional referral hospitals.

Zimbabwe (population 12.7 million) had a 2012 HIV prevalence of 15% among those aged 15–49 [15]. At the time of the baseline study, end 2011, 476,000 HIV-positive individuals were alive and on treatment [33] and ART provision was according to the 2010 WHO guidelines [2]. The health system in Zimbabwe is organized very similarly to Malawi with 4 tiers: a) *primary*: health clinics and rural hospitals run by primary care nurses with no resident doctor; b) *secondary*: district or general hospitals with a resident medical officer; c) *tertiary*: 7 provincial referral centres; and d) *quaternary*: 6 national central hospitals, connected by a referral system. There are provincial medical directorates responsible for coordinating districts within each province.

### Study Design

**Figure 1:**
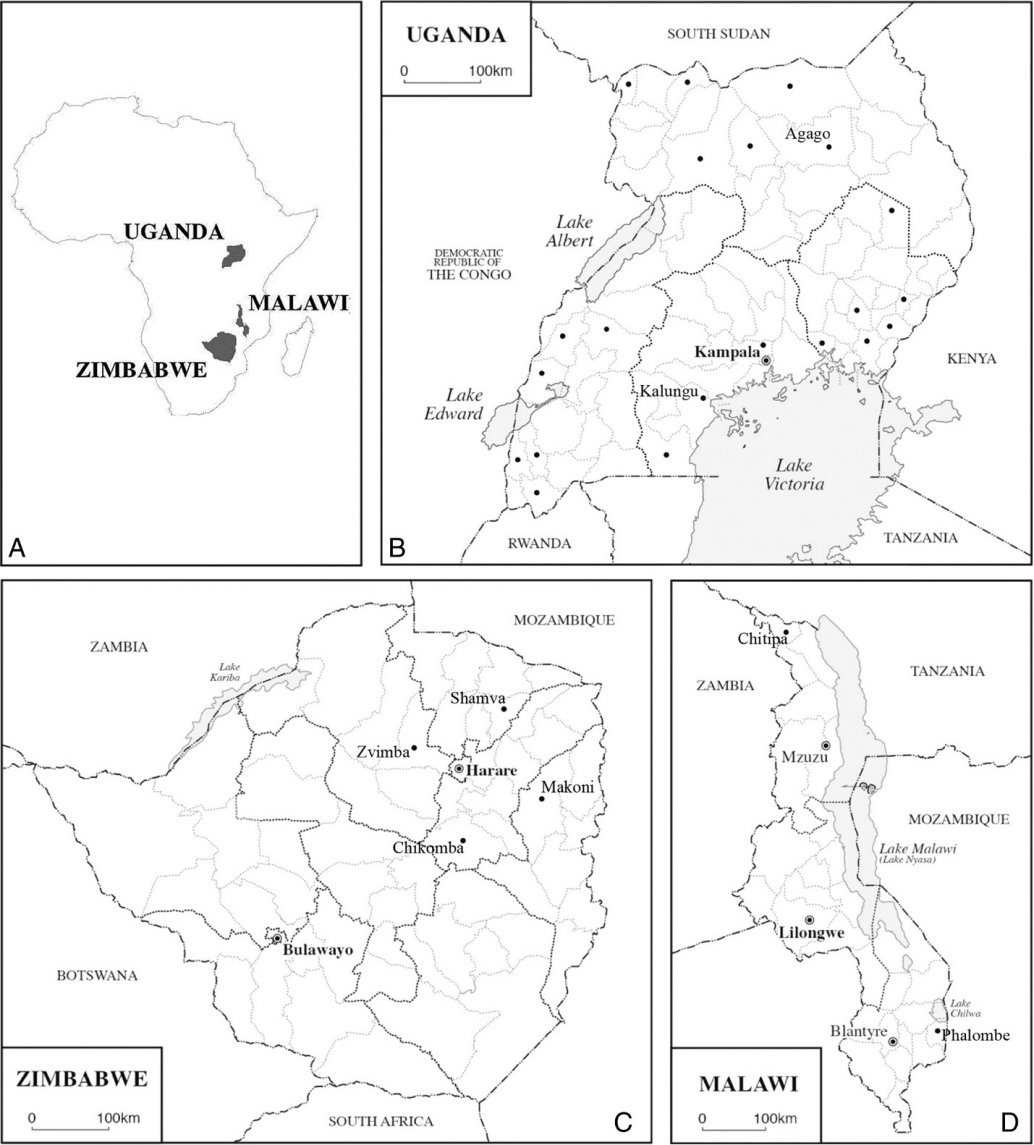
**Country Maps Demonstrating Geographic Locations of Cross Sectional Survey. A**. Locations of Malawi, Uganda and Zimbabwe in sub-Saharan Africa. **B**. Uganda: Dots indicate 22 districts included in the survey. 13 health facilities from 4 districts in the Central Region, 14 health facilities from 6 districts in the Northern Region, 6 health facilities from 6 districts in the Eastern Region and 6 health facilities from 6 districts in the Western Region were surveyed. Implementation project is taking place in Kalungu (Central Region) and Agago (Northern Region). **C**. Zimbabwe: 5 health facilities from Zvimba District (site of implementation project), 6 health facilities from Chikomba District, 3 health facilities from Shamva District, 6 health facilities from Makoni District and 2 health facilities in Harare were surveyed. **D**. Malawi: 3 health facilities from Chitipa (Northern Region), 3 health facilities from Lilongwe (Central Region) and 15 health facilities from Phalombe (Southern Region and site of implementation project) were surveyed.

A purposeful sample of 81 health facilities were selected in 3 regions of Malawi, 22 districts of Uganda and 4 districts in Zimbabwe, representing different geographical regions (Figure 1). The study sites were chosen to reflect a mix of rural, semi-urban and urban sites at the primary, secondary and tertiary health facility level. Facilities included those at different stages of ART provision (including primary care facilities with no ART provision) and areas at which the Lablite project [34] will be implemented. The facilities were reflective of the division of service provision in these settings between the Ministries of Health (MoHs) and private/mission facilities with services agreements with the public health system, as well as the different systems of health sector decentralization. Sites were selected with the direction of Ministries of Health at the national and provincial/district levels. Primary care facilities had very limited or no research links prior to Lablite.

A questionnaire (Additional file 1) was administered by a senior member of the study research team who had experience in conducting surveys and had a medical background. The study respondents were the in-charge staff of the facility, or a representative who was able to provide details on service provision and health care provider training at the site. Using mixed methods, the questionnaire was designed to capture information in 5 relevant areas: a) general facility description, overview of services provided and human resources for health (HRH) capacity, b) HIV testing and counselling (HTC) services, c) Prevention of Mother to Child Transmission of HIV (PMTCT) services, d) ART services, e) laboratory services. At the first visit to the facility an appropriate respondent who was fluent in English, was identified for each section of the questionnaire based on the best facility-level knowledge of the relevant area. No compensation was provided for participation in the study, and therefore the study investigators scheduled their data collection around the clinical responsibilities of the respondents. For section a) this was the nurse-in-charge or someone who had in-depth knowledge of human resources, inpatient and outpatient services and the referral system at the facility and community; for section b) this was an ART nurse/clinician/doctor; for section c) this was a PMTCT nurse or midwife; for section d) this was an ART nurse/clinician/doctor; and for section e) this was a laboratory technician.

Data collection in Malawi occurred in October 2011 (Southern Region) and March 2012 (Central and Northern Region), in Uganda in November 2011-April 2012, and in Zimbabwe in January-July 2012. In most facilities, data collection took 1 day, although, in some, data collectors returned for an additional day to collect information. Questions were related to service provision at the day of collection; information on stock-outs and numbers of CD4 tests were collected for the 3 months (quarter) prior to the interview date. For information regarding numbers of clients (e.g. ART patients and numbers of visits) interviewees referred to appropriate registers and records. For information regarding stock outs of HIV test kits and drugs, data were collected on the number of days in the 3 month period that the facility had no stock and interviewees referred to pharmacy records.

Descriptive statistical analysis was conducted on the cross-sectional data stratifying findings by health care system level using the definitions outlined above.

### Ethics statement

The study received ethical approval from the Malawi National Health Sciences Research Committee, the Joint Clinical Research Centre-Uganda Institutional Review Board/Research Ethics Committee, the Uganda National Council of Science and Technology, and the Medical Research Council of Zimbabwe. Informed written consent was obtained from the health care workers who completed the survey. The consent procedure was approved by the reviewing ethics committees/research boards and forms were translated into the local language as well as English. No individual-level patient data were collected.

## Results

### Characteristics of all health facilities included in the Lablite cross-sectional baseline survey

#### Description of health systems organization of all health facilities

In total, 53 primary care facilities (16 in Malawi, 21 in Uganda and 16 in Zimbabwe) and 25 secondary care facilities [3 in Malawi, 16 in Uganda (8 HC IVs and 8 district hospitals), 6 in Zimbabwe] were included. Tertiary referral centres at Kamuzu Central Hospital in Malawi and Kabale and Gulu Regional Referral Hospitals in Uganda were surveyed; no tertiary level site was surveyed in Zimbabwe (Table 1).

**Table 1:** **General characteristics and staffing in all health facilities included in the baseline survey**

	Malawi			Uganda			Zimbabwe	
	Primary (n = 16)	Secondary (n = 3)	Tertiary (n = 1)	Primary (n = 21)	Secondary (n = 16)	Tertiary (n = 2)	Primary (n = 16)	Secondary (n = 6)
Owner								
Public/MoH^1^	12 (75)	2 (66)	1 (100)	20 (95)	12 (75)	2 (100)	12 (75)	5 (83)
Private not for profit^2^	4 (25)	1 (33)	0 (0)	1 (5)	4 (25)	0 (0)	4 (25)	1 (17)
Charges for out-patient consultations								
No	12 (75)	2 (66)	1 (100)	21 (100)	11 (69)	2 (100)	4 (25)	0 (0)
Yes	4 (25)	1 (33)	0 (0)	0 (0)	5 (31)	0 (0)	12 (75)	6 (100)
Location								
Urban	2 (13)	1 (33)	1 (100)	3 (14)	4 (25)	2 (100)	2 (13)	1 (17)
Peri-urban	3 (19)	1 (33)	0 (0)	3 (14)	6 (38)	0 (0)	1 (6)	0 (0)
Rural	11 (69)	1 (33)	0 (0)	15 (71)	6 (38)	0 (0)	13 (81)	5 (83)
Access road								
Tarmac	3 (19)	1 (33)	1 (100)	1 (5)	6 (38)	1 (50)	12 (75)	6 (100)
Functioning dirt road	13 (81)	2 (66)	0 (0)	20 (95)	9 (56)	1 (50)	4 (25)	0 (0)
Water transport	0 (0)	0 (0)	0 (0)	0 (0)	1 (6)	0 (0)	0 (0)	0 (0)
Catchment population: Median (range)	29,275 (11,074-240,000)	50,015 (29,721-1,897,168)	5,490,000	9,000 (325–210,000)	70,295 (2,400-500,000)	2,250,000 (1,500,000-3,000,000)	8,616 (3,122-113,000)	13,747 (9,184-298,495)
Time (hours) to nearest tertiary facility: Median (range)	1.9 (.33-6)	1.5 (.25-5)	-				1.5 (.33-3.5)	1.3 (.25-2)
Physicians	0 (0–0)	2.8 (1.5-4)		0 (0–0)	2 (1–4.5)	5.5 (5–6)	0 (0–0)	1 (1–1)
Clinical Officers/Medical Assistants	1 (1–2)	25 (11–40)		2 (1–2)	4.5 (2–8)	19 (8–29)	0 (0–0)	1 (0–1)
Midwives/Nurses	2.5 (1–4.5)	42 (34–49)	309	4 (3–6)	22 (13–68)	99 (59–138)	6 (2.5-14)	66 (46–82)
Counsellors	0 (0–0)	0 (0–0)		0 (0–0)	0 (0–1)	0 (0–0)	1 (1–1)	4 (3–4)
Laboratory technicians/assistants^3^	0 (0–0.5)	4.5 (3–6)		1 (0–1)	2 (1.8-3)	8.5 (8–9)	0 (0–0)	3 (2–4)
Auxiliary staff^4^	5 (3.5-7.5)	70 (40–101)		2 (0–3)	6 (0–8)	57 (13–100)	3 (3–9)	64 (52–93)
Community health workers	18 (11–22)	31 (21–40)		0 (0–0)	0 (0–3)	2 (0–4)	0 (0-.5)	0 (0–0)
Administrative staff	0 (0–2)	7 (3–11)		0 (0–1)	1 (.5-6)	10 (7–13)	0 (0–0)	13 (6–18)

Values are n (col%) or median (IQR) unless stated otherwise. Where numbers do not sum to total this is missing data; additional missing data were as follows: catchment population (1 primary facility Malawi), time to nearest tertiary facility (3 primary facilities Zimbabwe; not collected in Uganda), staffing data (one secondary facility in Malawi only had data on numbers of physicians, one secondary care facility in Malawi had no data on number of physicians; tertiary care facility in Malawi only had data on number of midwives/nurses).

^1^Ministry of Health, local government or Rural District Council (Zimbabwe).

^2^Christian health association, Catholic mission, other mission or City of Harare (Zimbabwe).

^3^Laboratory staff were laboratory technicians except in Malawi, where in primary facilities there were 6 laboratory technicians and 2 laboratory assistants and in Uganda where in primary facilities there were 15 laboratory technicians and 2 laboratory assistants, in secondary facilities there were 29 laboratory technicians, 3 laboratory assistants, 1 microscopist and 11 unspecified laboratory staff, and in tertiary facilities there were 12 laboratory technicians and 5 laboratory assistants.

^4^Auxiliary staff include pharmacy, radiology staff as follows: Malawi: primary: 2 pharmacy technicians; secondary: 2 pharmacy technicians, 2 radiology staff; Uganda: primary: 0; secondary: 1 pharmacist, 2 pharmacy assistants, 2 radiographers; tertiary: 2 pharmacy technicians, 2 radiographers; Zimbabwe: primary: 0; secondary: 2 pharmacists, 4 pharmacy technicians, 1 radiographer, 1 x-ray operator.

For non-ART related outpatient consultations, public sector facilities run by MoH did not charge users fees in Malawi, however all mission facilities run by the Christian Health Association of Malawi (CHAM) did (5/20). In Uganda only 5/39 facilities charged for outpatient consultations (2 public sector, 2 Christian Health Association and 1 mission-run facility), all in secondary care. Conversely, in Zimbabwe, only 4/22 facilities did not charge users fees for outpatients, all in primary care (3 public sector and 1 Catholic mission).

The majority of primary care facilities were rural and in Malawi and Uganda, most were served by dirt road as opposed to all-weather tarmac roads. Secondary care facilities included a mixture of rural, peri-urban and urban facilities. All tertiary centres were urban. Median (range) reported time to travel to the nearest tertiary level facility by vehicle from primary and secondary level referring facilities was 1.8 hours (0.25-6) in Malawi and 1.5 hours (0.25-3.5) in Zimbabwe (data not available in Uganda). The catchment population of these facilities had wide variation, between and within country; primary care facilities on average served larger catchment populations in Malawi than in Uganda or Zimbabwe and urban primary care facilities were more likely to serve larger populations than rural or peri-urban centres.

Staffing at primary level facilities was predominantly comprised of nurses/midwives, although all but one facility in Malawi and Uganda were led by a non-physician clinical officer/medical assistant. Of note, in Malawi where there has been formalization of a community health worker cadre, known as the health surveillance assistant (HSA), 57% of non-administrative clinic staff in primary care were HSAs compared to 2% in Uganda and 12% in Zimbabwe, with most facilities in Uganda and Zimbabwe having no health care workers of this cadre. Only 6/16, 14/21 and 1/16 primary care facilities in Malawi, Uganda and Zimbabwe respectively had a laboratory technician or assistant, whereas most higher level facilities had several laboratory staff. Low numbers of administrative staff at the primary care level means administrative tasks fall to the frontline clinical staff; in Malawi, Uganda and Zimbabwe, 7/16 and 6/21 and 3/16 primary care sites had administrative staff. There were more administrative staff in secondary and tertiary level facilities.

#### Availability of clinical and laboratory services at all health facilities

Primary care facilities in all three countries offered basic care to patients including outpatient adult and paediatric medical care, family planning, antenatal care, and labour and delivery services (Table 2). All offered HTC and the majority provided PMTCT and cotrimoxazole preventive therapy (CPT) for HIV-positive patients. Most provided on-going TB treatment although in Malawi initiation of TB treatment may be in secondary care. They provided referrals for TB care to higher level health facilities as appropriate. Isoniazid preventive therapy (IPT) for HIV-positive patients was implemented at 38%, 29% and 6% of all primary facilities in Malawi, Uganda and Zimbabwe, respectively.

**Table 2:** **Provision of services** ***on-site*** **in all health facilities included in the baseline survey**

	Malawi			Uganda			Zimbabwe	
	Primary (n = 16)	Secondary (n = 3)	Tertiary (n = 1)	Primary (n = 21)	Secondary (n = 16)	Tertiary (n = 2)	Primary (n = 16)	Secondary (n = 6)
**Clinical services**								
Adult medical care	16 (100)	3 (100)	1 (100)	21 (100)	16 (100)	2 (100)	15 (94)	6 (100)
Paediatric medical care	16 (100)	3 (100)	1 (100)	20 (95)	16 (100)	2 (100)	14 (88)	6 (100)
Antenatal care	15 (94)	3 (100)	1 (100)	21 (100)	16 (100)	2 (100)	16 (100)	6 (100)
Obstetric care	12 (75)	3 (100)	1 (100)	18 (86)	16 (100)	2 (100)	16 (100)	6 (100)
Family planning	13 (81)	2 (67)	1 (100)	21 (100)	15 (94)	2 (100)	15 (100)	5 (83)
TB treatment	16 (100)	3 (100)	1 (100)	17 (81)	16 (100)	2 (100)	14 (88)	6 (100)
HIV testing and counselling	16 (100)	3 (100)	1 (100)	21 (100)	16 (100)	2 (100)	16 (100)	6 (100)
PMTCT	15 (94)	3 (100)	1 (100)	21 (100)	16 (100)	2 (100)	16 (100)	6 (100)
DNA PCR for infant diagnosis (off site)^1^	9 (82)	3 (100)	1 (100)	14 (67)	13 (81)	2 (100)	13 (100)	5 (100)
Cotrimoxazole prophylaxis for HIV positive patients	15 (94)	3 (100)	1 (100)	19 (90)	16 (100)	2 (100)	16 (100)	6 (100)
Isoniazid preventative therapy for HIV positive patients	6 (38)	1 (33)	1 (100)	6 (29)	6 (38)	2 (100)	1 (6)	3 (50)
Antiretroviral therapy	9 (56)	3 (100)	1 (100)	6 (29)	16 (100)	2 (100)	15 (94)	6 (100)
**Laboratory services**								
Haemoglobin	6 (38)	3 (100)	1 (100)	9 (43)	16 (100)	2 (100)	2 (13)	5 (83)
Malaria diagnosis (rapid or microscopy)	8 (50)	3 (100)	1 (100)	16 (76)	16 (100)	2 (100)	13 (81)	5 (83)
Sputum microscopy for TB	6 (38)	2 (67)	1 (100)	15 (71)	16 (100)	2 (100)	1 (6)	6 (100)
CSF microscopy	0 (0)	3 (100)	1 (100)	2 (10)	10 (63)	2 (100)	1 (6)	4 (67)
Cryptococcal diagnosis (Ag or microscopy)	0 (0)	3 (100)	1 (100)	2 (10)	11 (69)	2 (100)	1 (6)	6 (100)
Blood culture	0 (0)	1 (33)	1 (100)	0 (0)	1 (6)	0 (0)	0 (0)	3 (50)
Chest x-ray	1 (6)	3 (100)	1 (100)	0 (0)	9 (56)	2 (100)	0 (0)	4 (67)

Values are n (col %).

^1^Missing data for some facilities means that the denominators were not 100% of facilities in all columns for infant diagnosis. Percentages are of non-missing data.

At the time of the survey, decentralization of ART was incomplete: 9/16 primary care facilities in Malawi, 6/21 in Uganda and 15/16 in Zimbabwe had on-site ART provision. In all but one facility in Zimbabwe ART provision at the primary health level was conducted by mobile outreach teams from referral centres, as opposed to existing health facility staff. All secondary and tertiary care facilities provided ART. Early infant diagnosis through dry blood spots (DBS) DNA-PCR programming was reported as accessible offsite by referral in 9/11 (5 sites missing data), 14/21 and 13/13 (3 sites missing data) primary care sites in Malawi, Uganda and Zimbabwe respectively.

Simple on-site laboratory tests (Table 2) were more likely to be provided at the primary level in Uganda [haemoglobin 9/21 (43%) facilities; malaria testing 16/21 (76%); sputum microscopy for TB 15/21 (71%)] than in Malawi [6/16 (38%); 8/16 (50%); 6/16 (38%)] or Zimbabwe [2/16 (13%), 13/16 (81%), 1/16 (6%)]. One secondary care facility in Malawi did not have TB sputum microscopy and one secondary care site in Zimbabwe did not measure haemoglobin on-site, otherwise these tests were available in secondary and tertiary care. Cryptococcal diagnosis (antigen testing or India ink) was seldom available in primary care [0/16 (0%), 2/21 (10%) and 1/16 (6%) facilities in Malawi, Uganda and Zimbabwe] but mostly available in secondary care (all facilities in Malawi and Zimbabwe and 11/16 (69%) facilities in Uganda) and always in tertiary care. Microbiology (blood culture) was not readily available other than at 1 secondary and 1 tertiary referral hospital in Malawi (both through an academic research partnership), 1 secondary referral hospital in Uganda and 3 secondary level facilities in Zimbabwe. Only one primary care facility provided chest radiography on-site (in Malawi). In secondary care, chest radiography was available in 3/3 (100%), 9/16 (56%) and 4/6 (67%) facilities in Malawi, Uganda and Zimbabwe. The three tertiary care sites provided chest radiography.

#### Availability of key commodities for routine HIV care in all health facilities

Table 3 outlines stock outs of key commodities in HIV care in all facilities. Across the items surveyed, ART drugs for adults were largely available most of the time at all levels. Stock-outs of paediatric ART were problematic in Uganda where 3 of the 6 primary care facilities with ART provision and 3/16 secondary care facilities reported a stock-out in the three months prior to survey. Uganda recorded stock-outs at all levels for TB drugs, antibiotics and HIV commodities. Drug stock levels for opportunistic infections or management of intercurrent illness were not assessed, beyond antibiotics.

**Table 3:** **Stock-outs of HIV-test-kits and drugs in all health facilities over three months prior to survey**

	Malawi			Uganda			Zimbabwe	
	Primary	Secondary	Tertiary	Primary	Secondary	Tertiary	Primary	Secondary
	(n = 16)	(n = 3)	(n = 1)	(n = 21)	(n = 16)	(n = 2)	(n = 16)	(n = 6)
**Antibiotics**								
No. facilities with stock-outs	5/16	1/3	0/1	17/21	6/16	2/2	4/16	0/6
No. days/90 per facility if >0	14,14,30,60,60	30		14,14,14,21,30,30,30,30,35,42,59,60,60,60,70,75,90	7,10,14,21,28,30	14,30	5,7,90,90	
**TB drugs**								
No. facilities with stock-outs	1/16	1/3	0/1	9/17	2/16	1/2	2/14	1/6
No. days/90 per facility if >0	2	30		7,14,14,15,30,31,60,60,90	31,90	30	3,60	14
**Anti-malarial drugs**								
No. facilities with stock-outs	2/16	0/3	0/1	3/21	2/16	0/2	1/16	0/6
No. days/90 per facility if >0	5,7			14,14,14	21,30		7	
**HIV test kits**								
No. facilities with stock-outs	10/16	1/3	0/1	5/21	4/16	1/2	2/16	0/6
No. days/90 per facility if >0	3,8,14,14,14, 18,23,25,30, 30	Days missing		3,14,30,30,30	5,7,21,90	1	4,7	
**Cotrimoxazole for HIV positive patients**								
No. facilities with stock-outs	11/15	0/3	0/1	8/19	4/16	0/2	1/16	1/6
No. days/90 per facility if >0	14,21,31,45,60,60,66,70,90,90,90			20,21,60,60,66,90,90,90	28,30,60,90		7	90
**ART for PMTCT**								
No. facilities with stock-outs	2/15	2/3	1/1	3/21	1/16	0/2	0/16	0/6
No. days/90 per facility if >0	14,60	7,90	62	2,14,60	31			
**Adult ART**								
No. facilities with stock-outs	0/9	1/3	0/1	0/6	0/16	0/2	1/1	1/6
No. days/90 per facility if >0		30					30	14
**Paediatric ART**								
No. facilities with stock-outs	2/9	0/3	0/1	3/6	3/16	0/2		2/6
No. days/90 per facility if >0	1,7			14,90,90	14,30,60			14,40

Where denominator for facilities with stock outs is less than total facilities it is because the facility does not provide the relevant service. Additionally, for facilities in Zimbabwe providing ART, stock-outs are only applicable where the provision is static as opposed to outreach.

Stock outs of HIV test kits were noted in all three countries and, in Uganda, across all levels of care. Recent stock-outs of HIV-testing kits were not only frequent (Malawi: 55%, Uganda: 26%, Zimbabwe: 9% across all facility levels), but they often lasted for long periods of time (median days out of stock over 3 months were 16, 18 and 6 in Malawi, Uganda and Zimbabwe respectively). Similarly, although cotrimoxazole prophylaxis for HIV-positive patients was provided in 80/81 facilities, stock-outs occurred in 58%, 32%, and 9% of all facilities in Malawi, Uganda and Zimbabwe respectively.

### Characteristics of health facilities with on-site ART provision available

#### ART service availability and utilization at health facilities

Decentralization of ART has been rolled out to the primary health facility level since 2005 in all three countries but was only available in a subset of primary care facilities in this sample (Table 4). In total, 13 facilities in Malawi had on site ART provision including 9 primary care facilities; corresponding numbers were 24 facilities (including 6 primary care) in Uganda and 21 facilities (including 15 primary care; one of the 15 did no ART initiation) in Zimbabwe. In Malawi and Uganda the number of days the ART clinic ran per week varied in both primary and secondary care. In Zimbabwe, ART provision in primary health centres was predominantly conducted by an outreach team visiting from a referral centre (every 2 weeks) as opposed to by existing health facility staff. In Malawi and Uganda all facilities with ART provision were able to initiate and follow-up children on ART; in Zimbabwe 3/15 primary care facilities (with any ART provision on site) did not initiate children on ART on-site and 1/15 did no paediatric follow-up. At only 2 sites was ART available at Maternity or ANC/PMTCT at the time of the study.

**Table 4:** **ART service provision and usage in health facilities providing ART on-site**

		Malawi			Uganda			Zimbabwe	
		Primary	Secondary	Tertiary	Primary	Secondary	Tertiary	Primary	Secondary
		(n = 9)	(n = 3)	(n = 1)	(n = 6)	(n = 16)	(n = 2)	(n = 15 ^1^)	(n = 6)
Provision start year^2^									
2003-04		0 (0)	0 (0)	1 (100)	0 (0)	3 (20)	2 (100)	0 (0)	0 (0)
2005-06		1 (11)	1 (33)	0 (0)	2 (33)	12 (80)	0 (0)	2 (13)	3 (50)
2007-08		3 (33)	2 (66)	0 (0)	2 (33)	0 (0)	0 (0)	4 (27)	2 (33)
2009-10		2 (22)	0 (0)	0 (0)	1 (17)	0 (0)	0 (0)	7 (47)	1 (17)
2011		3 (33)	0 (0)	0 (0)	1 (17)	0 (0)	0 (0)	2 (13)	0 (0)
Days service provided									
1/fortnight		0 (0)	0 (0)	0 (0)	0 (0)	0 (0)	0 (0)	14 (93)	0 (0)
1-2/week		8 (89)	2 (66)	0 (0)	3 (50)	4 (25)	0 (0)	0 (0)	1 (17)
3-4/week		1 (11)	0 (0)	0 (0)	1 (17)	3 (19)	1 (50)	0 (0)	0 (0)
5/week		0 (0)	1 (33)	1 (100)	2 (33)	9 (56)	1 (50)	1 (7)	5 (100)
Adults ≥15 on ART^3,4^		516 (241–886)	2610 (2,438-9,600)	17,453	189 (56–604)	576 (385–890)	1,968 (1,523-2,412)	203 (97–359)	2,800 (1,976-3,726)
Adults ≥15 initiating ART per month^4^		49 (30–55)	37 (25–478)	597	11 (7–19)	25 (12–34)	66 (35–97)	13 (6–20)	50 (40–100)
Children on ART^3,4^					22 (9–51)	44 (21–59)	230 (223–237)	19 (10–31)	203 (165–318)
Children initiating ART per month^4^		4 (1–7)	7 (5–23)	27	1.5 (1–5)	3 (2–5)	3.5 (2–5)	2.5 (1–4.5)	5 (3–10)

Values are n (col%) or median (IQR).

^1^One primary care facility in Zimbabwe had a static ART clinic but did no ART initiation for children < 10 (numbers of children initiating and followed up not included); 14 primary care facilities provided outreach clinics. 13/14 primary care facilities with outreach initiated adults on ART; 12/14 initiated children. 14/14 followed up adults, 13/14 followed up children.

^2^Where numbers do not sum to column total this is due to missing data, percentages are presented for non-missing data.

^3^Numbers on ART for Malawi are for adults and children combined (given in adults’ row). Numbers on ART missing for one primary care facility in Malawi.

^4^Malawian MoH data for quarter July-September 2011 were used for numbers on ART and for numbers initiating ART for 6 Phalombe facilities. Interview data were used for facilities outside Phalombe. Zimbabwean MOH data for July-September 2011 were used for numbers on ART and numbers initiating ART (except for one primary care facility where data used were for March-May 2012). Ugandan MoH data for quarter October-December 2011 were used for numbers on ART and numbers initiating ART (except for 3 primary care sites and 2 secondary care sites where interview data were used for numbers initiating).

The total patients (adults and children) on ART in a primary care facility were highest in Malawi and lower in Uganda and Zimbabwe; as expected larger numbers were generally treated in Health Centre Level IV facilities or hospitals. In facilities in Uganda and Zimbabwe with adult and paediatric patients on ART, children constituted 8% and 8% of all individuals on ART respectively; and 11% and 14% of new patient initiations. This compares with estimated proportions in need of 16% and 14% [15]. These breakdowns were not available for Malawi.

#### ART drug regimen utilization: Adults, Paediatric and PMTCT regimens

In terms of first line adult ART regimens, across all facilities with ART provision in Malawi half (6/12; 1/13 provided no information) at the time of survey had Tenofovir (TDF)-based regimens available; otherwise Stavudine (d4T)-based regimens were still being used as the standardized first-line regimen (Additional file 2: Table S1a). In Uganda d4T has been phased out, and sites at all levels are giving either Zidovudine (ZDV)- or TDF-based first-line regimens, the latter available mainly at secondary centres. In Zimbabwe, the picture was more similar to Malawi and both countries had ZDV-based ‘alternative’ first-line regimens available at about one third of facilities. Boosted-Lopinavir (LPV/r) was the second-line regimen available in all countries; one Ugandan tertiary site also had boosted-Atazanavir (ATV/r). Few primary health facilities had second line available on-site, whereas 3/4, 15/18, and 4/6 secondary and tertiary level facilities had second line drugs available on-site in Malawi, Uganda and Zimbabwe respectively (Additional file 2: Table S1a).

ZDV-based regimens were primarily used for first-line ART for children in Malawi at sites at all levels (10/13) and Uganda (21/24). In Zimbabwe, d4T-regimens were being used in primary care facilities but secondary facilities had changed to ZDV. First-line regimens including Abacavir (ABC) were only used in Uganda (5 secondary/tertiary facilities). TDF was available for children in 3 facilities in Uganda, presumably in adolescents. LPV/r was very rarely used as first-line for young children (only 2 facilities in Uganda; 1 facility in Zimbabwe). Where available, second-line ART was almost always LPV/r and the pattern of referral was similar to adults. In many sites it appeared that children were receiving adult as opposed to specific paediatric formulations (Additional file 2: Table S1b).

For PMTCT drug regimens, in Malawi TDF-based ART was available in 63% facilities, in tandem with the planned roll-out of the Option B + PMTCT strategy, with the remainder doing Option A. Most (75%) facilities reported using infant nevirapine (NVP), including those where TDF-based ART was being used. In Uganda and Zimbabwe, Option A was being used almost universally, with infants receiving liquid NVP prophylaxis during the period of risk (Additional file 2: Table S1c).

#### Availability of laboratory services for monitoring treatment toxicity at health facilities with on-site ART provision

**Figure 2:**
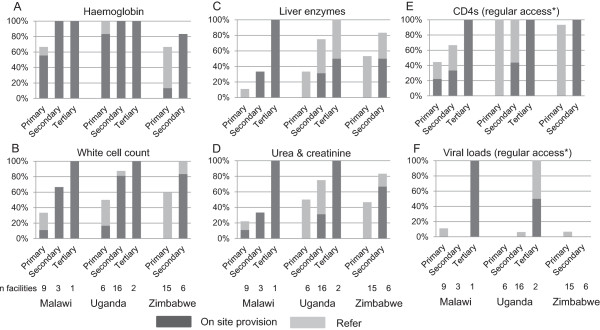
**Laboratory provision in health facilities providing ART on-site.** Proportion of facilities with on-site provision or able to refer for testing (remaining facilities have no provision). Referrals may be sample collection on-site with the sample sent to a reference laboratory or the patient may be referred to an alternative facility. **A**. Haemoglobin. **B**. White cell count. **C**. Liver enzymes. **D**. Urea and creatinine. **E**. CD4s. **F**. Viral loads. *Health facilities were asked whether or not they had regular access to CD4 and viral load testing: 1 additional facility in Malawi reported being able to refer for CD4; 3 , 15 and 1 additional facilities in Malawi, Uganda and Zimbabwe respectively reported they could refer for viral load testing but none did so regularly.

Primary and secondary level sites with ART provision were extremely limited with respect to on-site ability to provide laboratory testing for monitoring of side effects and treatment failure, in particular at the primary health care level (Figure 2). Haemoglobin was available on-site at 5/9, 5/6 and 2/15 primary care facilities, by up-referral in a further 1/9, 1/6 and 8/15 facilities in Malawi, Uganda and Zimbabwe respectively and not at all in the remainder. One secondary care facility in Zimbabwe did not provide haemoglobin but other secondary and all tertiary level facilities did so. White blood cells counts were only available on site in 2 primary care facilities (1 in Malawi and 1 in Uganda) and by referral in 2/9, 2/6 and 9/15 facilities. Most secondary care (2/3, 13/16 and 5/6) and all tertiary care facilities had on-site testing of full blood counts. In terms of basic biochemistry, liver function tests were less available and in primary care only 1/9, 2/6 and 8/15 facilities respectively could refer for tests (no on-site testing); numbers in secondary care were 1/3 (on-site), 12/16 (5 on-site) and 5/6 (4 on-site); 2/3 tertiary facilities had on-site testing (1 off site in Uganda). Access to urea and creatinine was similar.

#### Availability and utilization of CD4 and viral load testing for monitoring treatment effectiveness in health facilities providing ART

CD4 testing was available on-site at 2 primary care facilities (both in Malawi); other facilities collected samples on-site and transported them to reference laboratories (0/9 facilities in Malawi, 6/6 in Uganda, 13/15 in Zimbabwe), referred patients (2/9, 0/6, 1/15) or had no access to CD4s (5/9, 0/6, 1/15). Most secondary level facilities either had on-site CD4 testing (1/3, 7/16, 6/6) or collected samples on-site (0/3, 8/16, 0/6). The three tertiary level facilities all had on-site CD4 testing. CD4 testing prior to ART initiation was used in most secondary facilities, at least in selected patients, and, at primary level in 4/9 facilities in Malawi, 5/6 in Uganda and in 13/14 in Zimbabwe (1/15 primary care sites in Zimbabwe did follow-up post ART initiation only). In Malawi CD4-monitoring on ART was not used routinely in practice (as per national guidelines) [4], whereas in Uganda and Zimbabwe interviewees involved in ART provision in 5/6 and 10/14 primary care facilities respectively stated that the policy was to undertake 6-monthly CD4-monitoring on ART (missing data for one site in Zimbabwe); corresponding numbers for higher level facilities were 14/18 (including one facility with 3-monthly monitoring) and 2/6 (Table 5).

**Table 5:** **Reported use of CD4 and viral load testing in health facilities providing ART on-site**

		Malawi			Uganda			Zimbabwe	
		Primary	Secondary	Tertiary	Primary	Secondary	Tertiary	Primary	Secondary
		(n = 9)	(n = 3)	(n = 1)	(n = 6)	(n = 16)	(n = 2)	(n = 15)	(n = 6)
**CD4 testing regularly used prior to initiation**									
In all patients		0	1	1	1	4	0	9	2
Selected patients^1^		4	2	0	4	11	2	4	3
Not used regularly		5	0	0	1	1	0	1	0
**CD4 monitoring on ART in adults**									
Every 3 months		0	0	0	0	1	0	0	0
Every 6 months		0	0	0	5	11	2	10	2
Every 12 months		0	0	0	1	1	0	1	0
If clinically indicated		3	1	0	0	2	0	1	1
Not used regularly		6	2	1	0	1	0	2	1
**CD4 monitoring on ART in children**									
Every 3 months		0	0	0	0	1	0	0	0
Every 6 months		0	0	0	4	11	1	9	3
Every 12 months		0	0	0	1	0	1	0	0
If clinically indicated		3	1	0	0	2	0	1	1
Not used regularly		6	2	1	0	1	0	2	1
**Viral load monitoring on ART**									
If clinically indicated		1	0	1	0	1	2	1	0
Not used regularly		8	3	0	6	15	0	14	6

One primary care facility in Zimbabwe did no ART initiation. Two primary care facilities in Zimbabwe did not follow-up children. Otherwise, where numbers do not sum to total this is due to missing information.

^1^Four facilities in Uganda included as using CD4 testing prior to initiation in selected patients were doing CD4 testing in all adults but not in all children.

Data were available on numbers of CD4 tests provided (on or off site) over a 3-month period for 4 facilities providing ART (across all facility levels) in Malawi, 19 facilities in Uganda and 13 facilities in Zimbabwe (Table 6). On average the number of CD4 tests conducted per month was low given numbers of adults on ART and numbers of adults initiating ART. In Ugandan facilities where new patients initiating per month had stabilised to <5% adults on ART (so most CD4s would be used for monitoring) the ratio of numbers of CD4 tests to adults on ART translate into an estimated 4/12 facilities doing sufficient CD4s for 6-monthly monitoring of adults on ART, 3/12 for annual monitoring, 2/12 for 2-yearly monitoring and 3/12 for less frequent monitoring. Corresponding estimates for Zimbabwe were 0/9, 1/9, 2/9, and 6/9 (Table 6 and Additional file 2: Table S2).

**Table 6:** **CD4 tests performed per month in health facilities providing ART on-site with data available**

		Malawi			Uganda			Zimbabwe	
		Primary	Secondary	Tertiary	Primary	Secondary	Tertiary	Primary	Secondary
All facilities		(n = 2)	(n = 1)	(n = 1)	(n = 4)	(n = 14 ^$^)	(n = 1)	(n = 7)	(n = 6)
CD4s per month		84 (77–91)	57	831	40 (27–141)	82 (19–152)	388	10 (6–30)	171 (12–312)
Adults* on ART		201; missing	9,600	17,453	189 (74–705)	576 (336–874)	2,412	263 (129–1,845)	2,800 (1,976-3,726)
Adults initiating ART per month		46 (40–52)	478	597	11 (7–22)	27 (12–34)	35	13 (6–20)	50 (40–100)
Facilities where adults initiating ART per month <5% total adults* on ART		(n = 0)	(n = 1)	(n = 1)	(n = 2)	(n = 9^$$^)	(n = 1)	(n = 4)	(n = 5)
Estimated frequency of CD4s in adults* on ART**									
At least 6-monthly			0	0	2	2	0	0	0
Every 6 months to 1 year			0	0	0	2	1	0	1
Every 1 to 2 years			0	1	0	2	0	1	1
Less than once every 2 years			1	0	0	3	0	3	3

Values are median (IQR) or n.

Sample collection and testing was on-site at all facilities in Malawi, tertiary care facilities in Uganda and secondary care facilities in Zimbabwe. Samples were taken on-site and sent to a referral laboratory at all primary care facilities in Uganda and Zimbabwe.

^$^Sample collection and testing on-site (n = 7), samples taken on-site and sent to a referral laboratory (n = 6), patients sent to a referral laboratory (n = 1).

^$$^Sample collection and testing on-site (n = 4), samples taken on-site and sent to a referral laboratory (n = 4), patients sent to a referral laboratory (n = 1).

*Current adult patients at facility (except for Malawi where numbers are all ART patients).

**Estimated assuming constant number of patients on ART and constant rate of CD4-testing (includes all CD4s although some will be for initiation or in children). Of these facilities 0/1, 0/1 facilities in Malawi, 2/2, 6/9, 1/1 facilities in Uganda and 2/4, 2/5 facilities in Zimbabwe reported CD4-monitoring in adults was at least 6-monthly (across non-empty cells).

Only 2/13 (1 on-site), 3/24 (1 on-site) and 1/21 (off-site) facilities across all facility levels in Malawi, Uganda and Zimbabwe respectively had regular access to viral load testing and all testing was in selected patients, notably patients with signs of treatment failure (CD4 or weight loss) or patients with TB (Figure 2 and Table 5). Only one tertiary care facility in Malawi ever referred for resistance testing and it was to an academic research facility and not in the public sector.

## Discussion

The Lablite project teams, working with MoHs in Malawi, Uganda and Zimbabwe, conducted a comparative cross-sectional survey of HIV service provision in a sample of health facilities. This survey, carried out at the start of the Lablite Project during 2012, gives an overview of the operational realities for patients and frontline health workers at the facility level during a period where countries were in various stages of transitioning [2, 3, 31] from previous guidelines for ART provision to the WHO 2010 update [35].

In this multi-country survey, HIV services were available comprehensively within referral facilities. Primary health facility coverage of HTC and basic PMTCT services was also comprehensive, although stock-outs of HIV test-kits, and drugs would clearly have limited the services at times. Nevertheless, the findings show that coverage of ART and paediatric HIV services (in particular) to the decentralized health facility level remained incomplete even though roll-out to lower level primary health care facilities of ART had already been underway. At the time of the study, integration of ART services into ANC or Maternity was also not documented at any site.

The 2013 WHO Guidelines [22] advocating for adoption of Option B+ [36, 37] in many low resource, high prevalence operational settings necessitates that ART services become available and integrated into primary health centres where there are ANC and MCH services provided [38]. Early programmatic data shows considerable variation in retention on ART in Option B + women between health facilities [39, 40] as well as association of attrition with different approaches for timing of ART initiation relative to knowledge of HIV test result, place of ART initiation (antenatal or ART clinic), place of follow-up postnatally and post-breastfeeding, and type of support women receive [41]. As numbers of people on therapy are increasing, many health systems bottlenecks remain, in particular around inadequate support of human resources for health capacity, supply chain management and laboratory services infrastructure.

From a human resources perspective, successful ART roll-out relies on task shifting of clinical services in primary care facilities [42–45]. This study highlights further constraints beyond clinical tasks around laboratory technical capacity as well as administrative tasks. Malawi, which has one of the lowest professional health cadres-to-population ratios in the world, has uniquely adopted mitigation strategies which include the utilization of a formalized community health worker cadre, and use of NGO partners to implement a standardized supportive supervision and mentorship program led by the MoH national program.

Despite maturity of national ART roll-out programs with many programs operating for nearly a decade or longer, stock-outs of HIV test kits and drugs remains a critical bottleneck to access [46, 47], and needs urgent attention in the light of wider access being strived for in the WHO 2013 guidelines update. Mechanisms to forecast drugs through quantification of retention in care is particularly challenging for alternative first line and second line regimens [48, 49]. The data from this study highlighted that even at the primary care level, due to policy transition (e.g. d4T phase-out, introduction of TDF, and moving from NVP to efavirenz (EFV), various first-line regimens were available. The complexity of movement of large quantities of people in and out of regimen categories can be problematic during the period of transition, as the number of regimens used becomes more complicated with the evolution of the WHO guidelines. Accurate forecasting and ensuring availability of drugs is of key importance as patients may be triaged and delayed from substituting treatment regimens due to stock-out threats. Patients who may meet clinical criteria to be on certain regimens may be triaged out based on need and limited quantities. Downstream, ‘on the ground’ decisions made due to limited resources will misclassify groups of people, and contribute to lack of sensitivity in traditional methods of forecasting (which are based on monitoring numbers of people retained on specific regimens at a cross sectional point in time).

In our study, frequent stock outs of drugs, particularly cotrimoxazole, were reported in Malawi and Uganda, although less so in Zimbabwe. Stock outs emphasize the need to prioritize development of stock management capacities centrally and at the frontlines of delivery. Planners need to acknowledge and respond to the potential impacts of expanding ART eligibility criteria without adequate resourcing for drugs.

Maintaining supply of HIV test kits seems to be particularly problematic [50]. Already prioritization and quantification can be affected by challenges in coordinating multipronged HTC strategies supporting both community based screening of asymptomatic individuals, and facility based testing through PITC. Expanded eligibility, without concomitant supply side support for HTC kit logistics may contribute to ongoing stock out issues for facility based PITC programs targeting the patients who are most likely to need it the most e.g. patients with severe immunodeficiency, TB/HIV patients, HIV+ pregnant women eligible for Option B or Option B+ strategies.

As with other literature [51], our study reinforces that laboratory testing for drug toxicity and treatment effectiveness monitoring is available in these African countries, but generally not by patients accessing care at lowest level health facilities. Even haemoglobin (a very basic laboratory test) was available in less than half of primary health facilities. With respect to treatment monitoring, fewer lower level facilities had CD4 testing on-site, and in Malawi, few facilities used CD4 to monitor HIV patients (recommended for pre-ART [4]). On average the number of CD4 tests conducted per month was lower than reported or recommended in National guidelines, given numbers of adults on and initiating ART, even in sites which had access to CD4 testing. Overall and particularly in Malawi and Zimbabwe, very few facilities had any access to viral load monitoring, which is highlighted as the preferred monitoring method in WHO 2013 guidelines. Very few facilities have been able to adopt the recommendations for use of viral load monitoring for treatment failure, suggesting that in the immediate future, reinforcement of good clinical decision-making seems to be the most practical approach for monitoring patients on ART.

Finally, it is clear that paediatric HIV services are lagging behind management of adults on ART, and our study reflects the low coverage of paediatric ART for those in need in sub-Saharan Africa. Roll-out of DBS-PCR, which is important for early infant diagnosis and treatment has only been modestly successful [52–55] despite the availability of this modality in many countries for the last 5 years. The availability of paediatric ART is also inconsistent, as we show that in Uganda, stock outs of paediatric medications are relatively common and occur more frequently than adult ART. The paucity of child-friendly formulations [56] in most national program supply chains should be acknowledged and calls for better harmonisation across adult and paediatric formulations. The current implementation of more aggressive PMTCT strategies should certainly lead to a decrease in paediatric HIV incidence, but until issues of infrastructure, health care worker capacity, and supply chain constraints are managed, gaps in paediatric HIV services will remain. Acceleration of ART roll-out to children is key and can be done without routine toxicity monitoring [18].

One limitation of this study is that the numbers were relatively small, but purposeful sampling allowed selection from different regions and levels of health care to allow us to capture within country and between country differences. An additional limitation is that sampling may not necessarily be generalizable as representation of each country was based on sites selected by the country MoH partners. In Uganda, health centre IIIs and health centre IVs were selected from all four regions of the country, thus results are most generalizable nationally. In Zimbabwe facilities were included in 1 district each of 4 provinces (all in the North-East), namely, Mashonaland East, West, Central and Manicaland. Two urban facilities in the capital city were included for comparison. In Malawi, 3 facilities in the Northern region (2 rural, 1 periurban referral hospital in Chitipa District), 3 facilities in the Central region (1 urban tertiary hospital, 2 periurban facilities in Lilongwe District) and all 14 health facilities in the Phalombe District Health Office in the Southern Region (1 referral hospital, and a mixture of mission and public primary health facilities) were selected thus some inter-regional variations may have been missed. Data collected applied to the day of interview or, for stock-outs of HIV test kits and drugs, to the 3 months prior to the interview. Whereas the most recent data may be most reliable, this approach cannot capture trends, seasonal variations or unusual fluctuations. However, overall this survey is likely to provide a reasonable snapshot of health facilities by country and by level at one point in time. Most data were collected by interview and it is possible that the interviewees provided answers with a social desirability bias, in the belief that funding might be forthcoming if they highlighted the shortages and needs in services. A final limitation is that some data were extracted from the existing MoH monitoring and evaluation tools for HIV, and there may be some concerns regarding accuracy and consistency of such operational data. In Malawi, where facility level is available electronically through the national M and E system, data was harmonized with the national data to confirm accuracy and generalizability.

In the context of the recent release of the 2013 WHO guidelines [22], we can anticipate that the variability in practice and challenges on the ground documented in this study, can be expected to continue to be implementation bottlenecks to operationalizing expanded eligibility criteria. Care should be taken to quantify economic and operational feasibility especially in high prevalence, resource-limited countries where the approach to treatment is standardized and not individualized by clinicians. This survey was undertaken before roll-out of the Option B-plus strategy and we plan to repeat it in 2014. As Malawi and Uganda have since started Option B-plus and Zimbabwe is planning to start shortly, we will have the opportunity to compare the effect on ART roll-out before and after adoption of Option B-plus, as well as across the three countries.

## Conclusions

At the time of this survey, provision of HTC and PMTCT services was comprehensive across facility levels in Malawi, Uganda and Zimbabwe, although limited by stock-outs of supplies at some facilities. Decentralization of ART to primary care was ongoing but incomplete, being furthest ahead in Malawi; in Zimbabwe, ART provision in primary care was mostly by outreach teams, and in Uganda, provision was limited at primary care level. We found clear evidence of task shifting of clinic services in Malawi, but laboratory services were very limited in primary care, reinforcing the importance of good clinical management of patients. Stock-outs of drugs, particularly cotrimoxazole, and HIV-test kits, were reported in a number of facilities particularly in Uganda and Malawi. As demand for ART increases with adoption of Option B + (lifelong ART for pregnant and breastfeeding HIV-positive women) and higher threshold for ART initiation in the WHO 2013 guidelines, challenges of service provision on the ground may well increase if the health service barriers identified in this survey are not addressed concurrently.

## Electronic supplementary material

Additional file 1: **Chan_Lablite Data Collection Tools Final.** (PDF 506 KB)

Additional file 2: **Chan_Supplementarytables.** (DOC 142 KB)

## Abbreviations

ART: Antiretroviral therapy
TB: Tuberculosis
HC: Health centre
MoH: Ministry of health
HTC: HIV testing and counselling
PMTCT: Prevention of mother to child transmission of HIV
HSA: Health surveillance assistant
CPT: Cotrimoxazole preventive therapy
IPT: Isoniazid preventive therapy
DBS: Dry blood spot
TDF: Tenofovir
ZDV: Zidovudine
LPV/r: Boosted lopinavir
ATV/r: Boosted atazanavir
NVP: Nevirapine
EFV: Efavirenz.
