# Supplementary material for: The Lablite project: A cross-sectional mapping survey of decentralized HIV service provision in Malawi, Uganda and Zimbabwe
Source: BMC Health Serv Res. 2014 Aug 19;14:352. doi: 10.1186/1472-6963-14-352 (PMC4148932; doi:10.1186/1472-6963-14-352)
Supplement: Supplementary file 1 — Additional file 1: Chan_Lablite Data Collection Tools Final. (PDF 506 KB) [file 12913_2014_3455_MOESM1_ESM.pdf]

Baseline Assessment Tool for Health Facility (Lab-lite Study)

Section A: **Health Centre Service Overview**

Coding purposes

1a.	Country site	
2a.	Name of health facility	
3a.	District	

4a.	Informed consent obtained from interviewee(s)	[1] Yes [2] No
	**If no, please obtain informed consent before proceeding, and start again.**	

5a.	Date of interview	
6a.	Start time of interview	

7a.	Name of interviewee 1		
8a.	Professional qualification of interviewee 1	[1] Specialist physician	[2] Generalist physician
		[3] Clinical officer	[4] Medical assistant
		[5] Trained midwife	[6] Professional nurse
		[7] Enrolled nursing assistant	[8] Enrolled nurse
		[9] Counsellor	[10] Nurse counsellor
		[11] Community health worker/health surveillance asst.	
		[12] Other *(specify)*	

9a.	Name of interviewee 2		☐ None
10a.	Professional qualification of interviewee 2	[1] Specialist physician	[2] Generalist physician
		[3] Clinical officer	[4] Medical assistant
		[5] Trained midwife	[6] Professional nurse
		[7] Enrolled nursing assistant	[8] Enrolled nurse
		[9] Counsellor	[10] Nurse counsellor
		[11] Community health worker/health surveillance asst.	
		[12] Other *(specify)*	

11a.	Name(s) of interviewer(s)	

PROCEED TO NEXT PAGE.

Section A: **Health Centre Service Overview**

Coding purposes

A1.	Location	[1] Urban [2] Peri-urban [3] Rural
A2.	Distance from nearest large city/town (km)	
	(minutes by vehicle)	
	Name of the nearest city/town	
A3.	Access road	[1] Tarmac road [2] Functioning dirt road<br>[3] Other *(describe below):*
A4.	Current water supply	[1] Mains supply [2] Bore hole<br>[3] Shallow well [4] No water supply<br>[5] Other *(describe):*
A5.	Current electricity supply	[1] Mains supply [2] Solar power<br>[3] No electricity [4] Other *(describe below):*
A6.	Health facility catchment population	
A7.	Health facility catchment area (km^2)	
A8.	Furthest distance in catchment area (km)	
A9.	Type of facility	[1] Central Hospital [2] District Hospital<br>[3] Rural Hospital [4] Private/Mission Hospital<br>[5] Private/Mission Clinic [6] Health Centre (no level)<br>[7] Health Centre Level III [8] Health Centre Level IV<br>[9] Dispensary [10] Other *(list below):*<br>______________________
A10.	What is the nearest tertiary care facility?	
A11.	How long does it take a patient to travel from this facility to the nearest tertiary care facility (District or Central Hospital, whichever is closer)? List in # of hours.	hours
A12.	State the typical mode of transportation for a patient to travel to the nearest tertiary care facilty.	
A13.	What organization owns this facility?	[1] Ministry of Health<br>[2] Christian Health Assosication<br>Name of church: ______________________________<br>[3] NGO<br>Name of NGO: ______________________________<br>[4] Other<br>Name and type: ______________________________

PROCEED TO NEXT PAGE.

Section A: **Health Centre Service Overview**

Coding purposes

A14.	List number of **FULL TIME** staff at this facility	
A15.	How many regular **FULL TIME** staff of the following types are present at this facility?	
	# specialist physician(s)	☐ None
	# generalist physician(s)	☐ None
	# clinical officer(s)	☐ None
	# medical assistant(s)	☐ None
	# trained midwife(s)	☐ None
	# nurse(s)	☐ None
	# nurse counsellors(s)	☐ None
	# counsellor(s)	☐ None
	# trained laboratory technician(s)	☐ None
	# community health worker(s)/HSA(s)	☐ None
	# statistical/administrative clerk(s)	☐ None
	# auxilliary staff	☐ None
	# *other (specify)*	
	# *other (specify)*	
	# *other (specify)*	

A16.	List number of **PART TIME** staff (including locums) at this facility	
A17.	How many regular **PART TIME** staff (including locums) of the following types are present at this facility?	
	# specialist physician(s)	☐ None
	# generalist physician(s)	☐ None
	# clinical officer(s)	☐ None
	# medical assistant(s)	☐ None
	# trained midwife(s)	☐ None
	# nurse(s)	☐ None
	# nurse counsellors(s)	☐ None
	# counsellor(s)	☐ None
	# trained laboratory technician(s)	☐ None
	# community health worker(s)/HSA(s)	☐ None
	# statistical/administrative clerk(s)	☐ None
	# auxilliary staff	☐ None
	# *other (specify)*	
	# *other (specify)*	
	# *other (specify)*	

A18.	How many ABSENTEE DAYS were there in the past **three months** for the following staff cadres at this site?	
	# specialist physician(s)	☐ None
	# generalist physician(s)	☐ None
	# clinical officer(s)	☐ None
	# medical assistant(s)	☐ None
	# trained midwife(s)	☐ None
	# nurse(s)	☐ None
	# nurse counsellors(s)	☐ None
	# counsellor(s)	☐ None
	# trained laboratory technician(s)	☐ None
	# community health worker(s)/HSA(s)	☐ None
	# statistical/administrative clerk(s)	☐ None
	# auxilliary staff	☐ None
	# *other (specify)*	
	# *other (specify)*	
	# *other (specify)*	

A19.	How many OVERTIME HOURS did staff in the following cadres work in the past **three months** at this site?	
	# specialist physician(s)	☐ None
	# generalist physician(s)	☐ None
	# clinical officer(s)	☐ None
	# medical assistant(s)	☐ None
	# trained midwife(s)	☐ None
	# nurse(s)	☐ None
	# nurse counsellors(s)	☐ None
	# counsellor(s)	☐ None
	# trained laboratory technician(s)	☐ None
	# community health worker(s)/HSA(s)	☐ None
	# statistical/administrative clerk(s)	☐ None
	# auxilliary staff	☐ None
	# *other (specify)*	
	# *other (specify)*	
	# *other (specify)*	

A20.	How many staff in the following cadres LEFT THEIR POST in the past **three months** at this site?	
	# specialist physician(s)	☐ None
	# generalist physician(s)	☐ None
	# clinical officer(s)	☐ None
	# medical assistant(s)	☐ None
	# trained midwife(s)	☐ None
	# nurse(s)	☐ None
	# nurse counsellors(s)	☐ None
	# counsellor(s)	☐ None
	# trained laboratory technician(s)	☐ None
	# community health worker(s)/HSA(s)	☐ None
	# statistical/administrative clerk(s)	☐ None
	# auxilliary staff	☐ None
	# *other (specify)*	
	# *other (specify)*	
	# *other (specify)*	

A21.	How many NEW staff in the following cadres started at this site in the past **three months** at this site?	
	# specialist physician(s)	☐ None
	# generalist physician(s)	☐ None
	# clinical officer(s)	☐ None
	# medical assistant(s)	☐ None
	# trained midwife(s)	☐ None
	# nurse(s)	☐ None
	# nurse counsellors(s)	☐ None
	# counsellor(s)	☐ None
	# trained laboratory technician(s)	☐ None
	# community health worker(s)/HSA(s)	☐ None
	# statistical/administrative clerk(s)	☐ None
	# auxilliary staff	☐ None
	# *other (specify)*	
	# *other (specify)*	
	# *other (specify)*	

Coding purposes

A22.	Is staff housing available at this facility?	[1] Yes [2] No
	If yes, list number of staff housing units available	☐ None
	List which staff is the housing provided to	a) ☐ None
		b) ☐ None
		c) ☐ None
	Describe quality of staff housing units	[1] Good [2] Reasonable [3] Unusable ☐ None

A23.	How many beds does this facility have by category (if applicable)?	
	Emergency	☐ None
	Maternity ward	☐ None
	Surgery	☐ None
	Medical	☐ None
	Pediatric	☐ None
	Other *(list)*	☐ None
	Other *(list)*	☐ None
	Other *(list)*	☐ None

A24.	How many **in-patient days** were there at this facility in the months listed below?	
	# in-patient days SEPTEMBER 2011	☐ None
	# in-patient days AUGUST 2011	☐ None
	# in-patient days JULY 2011	☐ None

A25.	How many **outpatient visits** were conducted at this facility in the months listed below?	
	# outpatient visits SEPTEMBER 2011	☐ None
	# outpatient visits AUGUST 2011	☐ None
	# outpatient visits JULY 2011	☐ None

	Does this facility provide or refer for any of the following services?	
A26.	**General medicine**	[1] Yes, provide service [2] No, refer [3] Don't know<br>If no, then list referral site:
A27.	**Pediatric medicine**	[1] Yes, provide service [2] No, refer [3] Don't know<br>If no, then list referral site:
A28.	**Surgical services**	[1] Yes, provide service [2] No, refer [3] Don't know<br>If no, then list referral site:
A29.	**Tuberculosis treatment**	[1] Yes, provide service [2] No, refer [3] Don't know<br>If no, then list referral site: ______________________________<br>If yes, is HIV testing routinely offered to patients on TB treatment?<br>[1] Yes, in same consultation [2] Yes, refer to other service<br>[3] Not offered routinely [4] Don't know

Coding purposes

A30.	**soniazid preventative therapy for HIV+ patients** (IPT)	[1] Yes, provide service [2] No, refer [3] Don't know<br>If no, then list referral site: ______________________________<br>If yes, what are the criteria for IPT initiation?
A31.	**Sexually transimitted infection care** (STI)	[1] Yes, provide service [2] No, refer [3] Don't know<br>If no, then list referral site: ______________________________<br>If yes, is HIV testing routinely offered to STI clients?<br>[1] Yes, in same consultation [2] Yes, refer to other service<br>[3] Not offered routinely [4] Don't know
A32.	**HIV testing and counselling** (HTC)	[1] Yes, provide service [2] No, refer [3] Don't know<br>If no, then list referral site:
A33.	**Prevention of mother to child transmission services** (PMTCT)	[1] Yes, provide service [2] No, refer [3] Don't know<br>If no, then list referral site:
A34.	**Basic non-ART HIV/AIDS care** (e.g. diarrhea, pneumonia management)	[1] Yes, provide service [2] No, refer [3] Don't know<br>If no, then list referral site:
A35.	**Complex non-ART HIV/AIDS care** (e.g. severe sepsis, meningitis management)	[1] Yes, provide service [2] No, refer [3] Don't know<br>If no, then list referral site:
A36.	**Cotrimoxazole preventative therapy** for HIV+ patients (CPT)	[1] Yes, provide service [2] No, refer [3] Don't know<br>If no, then list referral site: ______________________________<br>If yes, what are the criteria for CPT initiation?<br>If yes, how many days of stock out of CTX in past 3 mths?

	Does this facility provide or refer for any of the following services? *(continued from previous page)*	
A37.	**Antiretroviral treatment** (ART)	[1] Yes, provide service [2] No, refer [3] Don't know<br>If no, then list referral site:
A38.	**Palliative care for AIDS** (e.g. opioid treatment)	[1] Yes, provide service [2] No, refer [3] Don't know<br>If no, then list referral site:

Coding purposes

A39.	**Antenatal care**	[1] Yes, provide service [2] No, refer [3] Don't know<br>If no, then list referral site: ______________________________<br>If yes, then record the average # of new ANC clients per month: __________________________<br>If yes, then list the routine laboratory testing offered to ANC clients:<br>[1] HIV test [2] Haemoglobin [3] Malaria test<br>[4] Syphillis test [5] No routine tests [6] Don't know<br>[7] Others *(specify)* : _________________________________<br>_________________________________
A40.	**Obstetric care** (L&D)	[1] Yes, provide service [2] No, refer [3] Don't know<br>If no, then list referral site:<br>If yes, then list average # of deliveries per month:
A41.	**Family planning**	[1] Yes, provide service [2] No, refer [3] Don't know<br>If no, then list referral site:
A42.	**Laboratory services**	[1] Yes, provide service [2] No, refer [3] Don't know<br>If no, then list referral site:
	Please list any other services or clinics provided at this facility below:	
A43.	**Other service or clinic** *(specify)*	[1] Yes, provide service [2] No, refer<br>If no, then list referral site:
A45.	**Other service or clinic** *(specify)*	[1] Yes, provide service [2] No, refer<br>If no, then list referral site:
A46.	**Other service or clinic** *(specify)*	[1] Yes, provide service [2] No, refer<br>If no, then list referral site:
A47.	Does this facility charge for outpatient consultations?<br>*If yes, describe the costs and amounts.*	[1] Yes [2] No
A48.	Does this facility charge for in-patient stays?<br>*If yes, describe the costs and amounts.*	[1] Yes [2] No
A49.	Does this facility charge for drugs (medications)?<br>*If yes, describe the costs and amounts.*	[1] Yes [2] No

Coding purposes

A50.	Does this facility charge for laboratory tests? *If yes, describe the costs and amounts.*	[1] Yes [2] No
A51.	Aside from transportation and food, are there any other medical costs that patients encounter at your facility? *If yes,please list & describe the costs.*	[1] Yes [2] No<br>a) ____________________<br>b) ____________________<br>c) ____________________<br>d) ____________________
A52.	Any other research team(s) working at this facility? *If yes, which research team(s)?*	[1] Yes [2] No<br>a) ____________________<br>b) ____________________<br>c) ____________________
A53.	What is the **main** funder for the following items at your facility?	
	Buildings and infrastructure	[1] Government [2] NGO<br>[3] Private institution [4] Research institution/University<br>[5] Faith-based provider [6] Don't know<br>[7] Other ____________________
	Maintenance of buildings and infrastructure	[1] Government [2] NGO<br>[3] Private institution [4] Research institution/University<br>[5] Faith-based provider [6] Don't know<br>[7] Other ____________________
	Staff salaries	[1] Government [2] NGO<br>[3] Private institution [4] Research institution/University<br>[5] Faith-based provider [6] Don't know<br>[7] Other ____________________
	ART drugs	[1] Government [2] NGO<br>[3] Private institution [4] Research institution/University<br>[5] Faith-based provider [6] Don't know<br>[7] Other ____________________

Coding purposes

A54.	Please state the # of days of stock outs of the following items at this facility in the past 3 months	
	TB drugs	☐ None
	Antibiotics	☐ None
	Antimalarial drugs	☐ None
	Other *(specify)*	
	Other *(specify)*	
	Other *(specify)*	

A55.	Using words and/or pictures, please describe the referral system at this facility. Please include all places patients, clients and laboratory specimens that are referred to your facility from other places and people, and all the places and people that your facility refers patients, clients and laboratory specimens to.

A56. Please describe the patient flow for all the client/patient services discussed (use words and/or pictures)

A57.	Please make arrangements to get patient register data for the following services, if applicable:		
	TB register	[1] Received [2] Not received [3] Not applicable	
	PMTCT register	[1] Received [2] Not received [3] Not applicable	
	HTC register	[1] Received [2] Not received [3] Not applicable	
	TB register	[1] Received [2] Not received [3] Not applicable	
	ART register	[1] Received [2] Not received [3] Not applicable	
	Pre-ART register	[1] Received [2] Not received [3] Not applicable	
	TB-HIV register	[1] Received [2] Not received [3] Not applicable	
	ANC register	[1] Received [2] Not received [3] Not applicable	
	L&D register	[1] Received [2] Not received [3] Not applicable	

A58.	End time of this section	

PLEASE PROCEED TO NEXT SECTION (SECTION B)

Section B: **HIV Testing and Counselling**

Coding purposes

1b.	Country site	
2b.	Name of health facility	
3b.	District	

4b.	Informed consent obtained from interviewee(s)	[1] Yes [2] No
	**If no, please obtain informed consent before proceeding, and start again.**	

5b.	Date of interview	
6b.	Start time of interview	

7b.	Name of interviewee 1		
8b.	Professional qualification of interviewee 1	[1] Specialist physician	[2] Generalist physician
		[3] Clinical officer	[4] Medical assistant
		[5] Trained midwife	[6] Professional nurse
		[7] Enrolled nursing assistant	[8] Enrolled nurse
		[9] Counsellor	[10] Nurse counsellor
		[11] Community health worker/health surveillance asst.	
		[12] Other *(specify)*	

9b.	Name of interviewee 2		☐ None
10b.	Professional qualification of interviewee 2	[1] Specialist physician	[2] Generalist physician
		[3] Clinical officer	[4] Medical assistant
		[5] Trained midwife	[6] Professional nurse
		[7] Enrolled nursing assistant	[8] Enrolled nurse
		[9] Counsellor	[10] Nurse counsellor
		[11] Community health worker/health surveillance asst.	
		[12] Other *(specify)*	

11b.	Name(s) of interviewer(s)	

PROCEED TO NEXT PAGE.

Section B: **HIV Testing and Counselling**

Coding purposes

B1.	Are HTC services available at this facility?	[1] Yes [2] No -> if no, skip to SECTION C.
B2.	**If yes,** start date of HTC services at this facility?	MM YYYY ☐ Don't know
B3.	What is the schedule of HTC services at this facilty? *List the hours for each day of the week that HTC services are available*	Mon:<br>Tues:<br>Wed:<br>Thurs:<br>Fri:
B4.	How many HTC visits were conducted at this facility in the months listed below?	
	# HTC visits SEPTEMBER 2011	☐ None
	# HTC visits AUGUST 2011	☐ None
	# HTC visits JULY 2011	☐ None
B5.	List the number of staff that provide HTC as a part of their ***regular duties*** at this facility, by provider type:	
	# Physician(s)	☐ None
	# Clinical officer(s)	☐ None
	# Medical assistant(s)	☐ None
	# Midwife(s)	☐ None
	# Nurse(s)	☐ None
	# Nurse counsellor(s)	☐ None
	# Dedicated counsellor(s)	☐ None
	# Community health worker/HSA(s)	☐ None
	# *Other (specify)*	
	# *Other (specify)*	
	# *Other (specify)*	
B6.	List the number of staff that ***have been trained*** to provide HTC at this facility, by provider type:	
	# Physician(s)	☐ None
	# Clinical officer(s)	☐ None
	# Medical assistant(s)	☐ None
	# Midwife(s)	☐ None
	# Nurse(s)	☐ None
	# Nurse counsellor(s)	☐ None
	# Dedicated counsellor(s)	☐ None
	# Community health worker/HSA(s)	☐ None
	# *Other (specify)*	
	# *Other (specify)*	
	# *Other (specify)*	
B7.	What is the HIV test strategy for adults and children aged >18 months at this facility? *Select all that apply.*<br>[1] Rapid HIV test(s) [2] ELISA [3] Western Blot [4] PCR [5] Don't know [6] Other *(specify below)*<br>______________________	
B8.	#Days stock-out of HIV test kits in past 3 months	☐ None

Coding purposes

B9.	Describe how community groups, including CBOs, are involved in HTC provision and support at this facility.	

B10.	What are the challenges, if any, that health providers face in providing HTC at this facility?

a)
b)
c)
d)
e)

B11.	Other important information about HTC at this site.

B12.	End time of this section	

PLEASE PROCEED TO NEXT SECTION (SECTION C)

Coding purposes

1c.	Country site	
2c.	Name of health facility	
3c.	District	

4c.	Informed consent obtained from interviewee(s)	[1] Yes [2] No
	**If no, please obtain informed consent before proceeding, and start again.**	

5c.	Date of interview	
6c.	Start time of interview	

7c.	Name of interviewee 1	
8c.	Professional qualification of interviewee 1	[1] Specialist physician [2] Generalist physician<br>[3] Clinical officer [4] Medical assistant<br>[5] Trained midwife [6] Professional nurse<br>[7] Enrolled nursing assistant [8] Enrolled nurse<br>[9] Counsellor [10] Nurse counsellor<br>[11] Community health worker/health surveillance asst.<br>[12] Other *(specify)*

9c.	Name of interviewee 2	☐ None
10c.	Professional qualification of interviewee 2	[1] Specialist physician [2] Generalist physician<br>[3] Clinical officer [4] Medical assistant<br>[5] Trained midwife [6] Professional nurse<br>[7] Enrolled nursing assistant [8] Enrolled nurse<br>[9] Counsellor [10] Nurse counsellor<br>[11] Community health worker/health surveillance asst.<br>[12] Other *(specify)*

11c.	Name(s) of interviewer(s)	

PROCEED TO NEXT PAGE.

Section C: **PMTCT**

Coding purposes

C1.	Are PMTCT services available at this facility?	[1] Yes [2] No -> if no, skip to SECTION D.
C2.	**If yes,** start date of PMTCT services at this facility?	MM YYYY ☐ Don't know
C3.	What is the schedule of PMTCT services at this facilty? *List the hours for each day of the week that PMTCT services are available*	Mon:<br>Tues:<br>Wed:<br>Thurs:<br>Fri:
C4.	How many new clients were enrolled in PMTCT at this facility in the months listed below?	
	# new PMTCT clients enrolled SEPTEMBER 2011	☐ None
	# new PMTCT clients enrolled AUGUST 2011	☐ None
	# new PMTCT clients enrolled JULY 2011	☐ None
C5.	List the number of staff that provide PMTCT as a part of their ***regular duties*** at this facility, by provider type:	
	# Physician(s)	☐ None
	# Clinical officer(s)	☐ None
	# Medical assistant(s)	☐ None
	# Midwife(s)	☐ None
	# Nurse(s)	☐ None
	# Nurse counsellor(s)	☐ None
	# Dedicated counsellor(s)	☐ None
	# Community health worker/HSA(s)	☐ None
	# *Other (specify)*	
	# *Other (specify)*	
C6.	List the number of staff that ***have been trained*** to provide PMTCT at this facility, by provider type:	
	# Physician(s)	☐ None
	# Clinical officer(s)	☐ None
	s	☐ None
	# Midwife(s)	☐ None
	# Nurse(s)	☐ None
	# Nurse counsellor(s)	☐ None
	# Dedicated counsellor(s)	☐ None
	# Community health worker/HSA(s)	☐ None
	# *Other (specify)*	
	# *Other (specify)*	
C7.	What is the standard PMTCT regimen available at this facility? *Specify the ARVs and dosing schedule.*	Mother:<br>Infant:
C8.	Is this the same PMTCT regimen as the national guideline?	[1] Yes [2] No [3] Don't know
C9.	# Days stock-out of ARVs for PMTCT in past 3 mths	☐ None

Coding purposes

C10.	Is cotrimoxazole prophylaxis provided to HIV+ pregnant mothers?	[1] Yes [2] No [3] Don't know
C11.	Is cotrimoxazole prophylaxis provided to exposed infants?	[1] Yes [2] No [3] Don't know
C12.	# Days stock-out of cotrimoxazole prophylaxis (CTP)	☐ None

C13. Is infant replacement feeding regularly available for HIV+ mothers who desire it?

[1] Yes, provide service at this facility [2] Yes, available at a referral facility [3] Not available [4] Don't know

C14. Is elective C-section regularly available for HIV+ motherts who desire it?

[1] Yes, provide service at this facility [2] Yes, available at a referral facility [3] Not available [4] Don't know

C15. What is the HIV test strategy for ***infants*** aged ≤18 months at this facility? *Select all that apply.*

[1] Rapid HIV test(s) [2] ELISA [3] Western Blot [4] PCR (EID) [5] Don't know [6] Other ______________

C16.	Describe how HIV+ mothers are followed up and supported at this facility.	
C17.	Describe how exposed infants are followed up and supported at this facility.	
C18.	Describe how community groups, including CBOs, are involved in PMTCT provision and support at this facility.	
C19.	Describe how volunteers, including expert patients are involved in PMTCT provision and support at this facility.	

C20. What are the challenges, if any, that health providers face in providing PMTCT at this facility?

a)

b)

c)

d)

e)

Coding purposes

C21.	Other important information about PMTCT at this site (e.g. support groups, male involvement, etc.):

C22.	End time of this section	

PLEASE PROCEED TO NEXT SECTION (SECTION D)

Section D: **Antiretroviral Treatment**

Coding purposes

1d.	Country site	
2d.	Name of health facility	
3d.	District	

4d.	Informed consent obtained from interviewee(s)	[1] Yes [2] No
	**If no, please obtain informed consent before proceeding, and start again.**	

5d.	Date of interview	
6d.	Start time of interview	

7d.	Name of interviewee 1	
8d.	Professional qualification of interviewee 1	[1] Specialist physician [2] Generalist physician<br>[3] Clinical officer [4] Medical assistant<br>[5] Trained midwife [6] Professional nurse<br>[7] Enrolled nursing assistant [8] Enrolled nurse<br>[9] Counsellor [10] Nurse counsellor<br>[11] Community health worker/health surveillance asst.<br>[12] Other *(specify)*

9d.	Name of interviewee 2	☐ None
10d.	Professional qualification of interviewee 2	[1] Specialist physician [2] Generalist physician<br>[3] Clinical officer [4] Medical assistant<br>[5] Trained midwife [6] Professional nurse<br>[7] Enrolled nursing assistant [8] Enrolled nurse<br>[9] Counsellor [10] Nurse counsellor<br>[11] Community health worker/health surveillance asst.<br>[12] Other *(specify)*

11d.	Name(s) of interviewer(s)	

PROCEED TO NEXT PAGE.

Section D: **Antiretroviral Treatment**

Coding purposes

D1.	Are ART services available at this facility?	[1] Yes [2] No -> if no, consider applying ART readiness tool and skip to SECTION E.
D2.	**If yes,** start date of ART services at this facility?	MM YYYY ☐ Don't know
D3.	Where are ART services located at this facility?	[1] ART is provided in the general OPD<br>[2] ART is provided in a separate/dedicated clinic.<br>[3] Other (specify): ___________
D4.	What is the schedule of ART services at this facilty?<br>*List the hours for each day of the week and which ART services are available*	Mon:<br>Tues:<br>Wed:<br>Thurs:<br>Fri:
D5.	# ADULTS 15 and over currently on ART at this facility:	☐ Don't know
D6.	# CHILDREN <15 currently on ART at this facility:	☐ Don't know
D7.	Average # ADULTS 15 and over ***initiated*** on ART per month at this facility:	☐ None ☐ Don't know
D8.	Average # CHILDREN <15 ***initiated*** on ART per month at this facility:	☐ None ☐ Don't know
D9.	How many ART patient visits (for any reason) occured at this facility in the months listed below?	
	# ART patient visits SEPTEMBER 2011	☐ None
	# ART patient visits AUGUST 2011	☐ None
	# ART patient visits JULY 2011	☐ None
D10.	Is there integration of TB and ART services at this facility?	[1] TB & ART services are integrated (same provider does both)<br>[2] TB & ART services are co-located (different providers in same clinic)<br>[3] No [4] Don't kow
D11.	Is CD4 testing regularily available at this facility?	[1] Yes, sample collection and testing done on site<br>[2] Yes, sample collection done on site and sent to referral lab<br>[3] Yes, but patients are sent to a referral lab<br>[4] CD4 testing not regularily available
D12.	What is the standard first-line ART regimen for **adults** available at this facility?	
D13.	Is this the same ART regimen as the national guideline?	[1] Yes [2] No [3] Don't know
D14.	#Days stock-out of first-line ARVs for **adults** in past 3 mths?	☐ None
D15.	Are adverse drug reactions (side-effects) to first-line ART in **adults** managed at this facility?	[1] Yes [2] No -> If no, list referral site: ___________
D16.	Is an alternative first-line ART regimen available for **adults** at this facility?	[1] Yes [2] No -> If no, list referral site: ___________<br>*If yes, list the regimen.*
D17.	Is a second-line ART regimen available for **adults** at this facility?	[1] Yes [2] No -> If no, list referral site: ___________

		If yes, list the regimen.
D18.	What is/are the criteria used for ART initiation in **adults**? *Check all that apply.*	[1] WHO stage [2] CD4 count<br>[3] Don't know [4] Other: ________________________
D19.	Describe the schedule for clinical follow up in **adults** in the first 3 months of ART initiation.	
D20.	Describe the schedule for clinical follow up in **adults** after the first 3 months of ART initiation.	
D21.	What is the standard first-line ART regimen for **children** available at this facility?	☐ Not available, list referral site: ______________
D22.	Is this the same ART regimen as the national guideline?	[1] Yes [2] No [3] Don't know
D23.	#Days stock-out of first-line ARVs for **children** in past 3 mths?	☐ None
D24.	Are adverse drug reactions (side-effects) to first-line ART in **children** managed at this facility?	[1] Yes [2] No -> If no, list referral site: ______________
D25.	Is an alternative first-line ART regimen available for **children** at this facility?	[1] Yes [2] No -> If no, list referral site: ______________<br>*If yes, list the regimen.*
D26.	Is a second-line ART regimen available for **children** at this facility?	[1] Yes [2] No -> If no, list referral site: ______________<br>*If yes, list the regimen.*
D27.	What is/are the criteria used for ART initiation in **children**? *Check all that apply.*	[1] WHO stage [2] CD4 count<br>[3] Don't know [4] Other: ________________________
D28.	Describe the schedule for clinical follow up in **children** in the first 3 months of ART initiation.	
D29.	Describe the schedule for clinical follow up in **children** after the first 3 months of ART initiation.	
D30.	How is CD4 testing regularily utilized at this facility in patients prior to ART initiation?	[1] All adults prior to ART initiation<br>[2] All children prior to ART initiation<br>[3] Selected adults and children prior to ART initiation<br>*Describe selection criteria:* ___________________________

[4] CD4 testing not utilized in patients prior to ART initiation

D31.	How is CD4 testing regularly utilized at this facility in patients after ART initiation?<br><br>*Check all that apply.*	[1] Every 3 mths in adults [2] Every 3 mths in children<br>[3] Every 6 mths in adults [4] Every 6 mths in children<br>[5] Every year in adults [6] Every year in children<br>[7] Only when clinically indicated (i.e. when necessary)<br>[8] CD4 testing not regularly utilized<br>[9] Other (*list*): ___________________
D32.	Is viral load testing regularly available at this facility?	[1] Yes [2] No [3] Don't know<br>If yes, describe how viral load testing is utilized:
D33.	Is HIV resistance testing regularly available at this facility?	[1] Yes [2] No [3] Don't know<br>If yes, describe how HIV resistance testing is utilized:
D34.	How is patient adherence to ART monitored at this facility? *Check all that apply.*	[1] Self report [2] Pill count by health care provider<br>[3] Viral load [4] Don't know<br>[5] Other *(specify)* : ________________
D35.	Describe how patients on ART are supported for adherence at your facility.	
D36.	Describe how defaulter tracing is done at your facility.	

D37. List the # of staff that provide HIV care as a part of their ***regular duties*** , by provider type and their role:

Physician(s) # ___ [ ] Regular staff at this facility [ ] Visiting staff to this facility or part of mobile team
[ ] ART initiation [ ] ART routine follow-up [ ] ART complex care (side effects, 2nd line)
[ ] WHO staging [ ] Pediatric ART [ ] CPT and other non-ART HIV care

Clinical officer(s) # ___ [ ] Regular staff at this facility [ ] Visiting staff to this facility or part of mobile team
[ ] ART initiation [ ] ART routine follow-up [ ] ART complex care (side effects, 2nd line)
[ ] WHO staging [ ] Pediatric ART [ ] CPT and other non-ART HIV care

Medical asst(s) # ___ [ ] Regular staff at this facility [ ] Visiting staff to this facility or part of mobile team
[ ] ART initiation [ ] ART routine follow-up [ ] ART complex care (side effects, 2nd line)
[ ] WHO staging [ ] Pediatric ART [ ] CPT and other non-ART HIV care

Midwife(s) # ___ [ ] Regular staff at this facility [ ] Visiting staff to this facility or part of mobile team
[ ] ART initiation [ ] ART routine follow-up [ ] ART complex care (side effects, 2nd line)

[ ] WHO staging [ ] Pediatric ART [ ] CPT and other non-ART HIV care

Nurse(s) # ___ [ ] Regular staff at this facility [ ] Visiting staff to this facility or part of mobile team
[ ] ART initiation [ ] ART routine follow-up [ ] ART complex care (side effects, 2nd line)
[ ] WHO staging [ ] Pediatric ART [ ] CPT and other non-ART HIV care

Other ( ) # ___ [ ] Regular staff at this facility [ ] Visiting staff to this facility or part of mobile team
[ ] ART initiation [ ] ART routine follow-up [ ] ART complex care (side effects, 2nd line)
[ ] WHO staging [ ] Pediatric ART [ ] CPT and other non-ART HIV care

D38. List the number of staff that ***have been trained*** to provide adult HIV care at this facility, by provider type and role:

Physician(s) # ___ [ ] Regular staff at this facility [ ] Visiting staff to this facility or part of mobile team
[ ] ART initiation [ ] ART routine follow-up [ ] ART complex care (side effects, 2nd line)
[ ] WHO staging [ ] Pediatric ART [ ] CPT and other non-ART HIV care

Clinical officer(s) # ___ [ ] Regular staff at this facility [ ] Visiting staff to this facility or part of mobile team
[ ] ART initiation [ ] ART routine follow-up [ ] ART complex care (side effects, 2nd line)
[ ] WHO staging [ ] Pediatric ART [ ] CPT and other non-ART HIV care

Medical asst(s) # ___ [ ] Regular staff at this facility [ ] Visiting staff to this facility or part of mobile team
[ ] ART initiation [ ] ART routine follow-up [ ] ART complex care (side effects, 2nd line)
[ ] WHO staging [ ] Pediatric ART [ ] CPT and other non-ART HIV care

Midwife(s) # ___ [ ] Regular staff at this facility [ ] Visiting staff to this facility or part of mobile team
[ ] ART initiation [ ] ART routine follow-up [ ] ART complex care (side effects, 2nd line)
[ ] WHO staging [ ] Pediatric ART [ ] CPT and other non-ART HIV care

Nurse(s) # ___ [ ] Regular staff at this facility [ ] Visiting staff to this facility or part of mobile team
[ ] ART initiation [ ] ART routine follow-up [ ] ART complex care (side effects, 2nd line)
[ ] WHO staging [ ] Pediatric ART [ ] CPT and other non-ART HIV care

Other ( ) # ___ [ ] Regular staff at this facility [ ] Visiting staff to this facility or part of mobile team
[ ] ART initiation [ ] ART routine follow-up [ ] ART complex care (side effects, 2nd line)
[ ] WHO staging [ ] Pediatric ART [ ] CPT and other non-ART HIV care

D39.	Where are antiretroviral medications dispensed for patients on ART at this facility?	[1] Directly in ART clinic [2] Pharmacy on-site<br>[3] Pharmacy off-site [4] Don't know<br>[5] Other *(specify)* : ________________________
D40.	Describe how community groups, including CBOs, are involved in ART provision and support at this facility.	
D41.	Describe how volunteers, including expert patients are involved in ART provision and support at this facility.	

D42. What are the challenges, if any, that health providers face in providing ART at this facility?
a)
b)
c)

Coding purposes

d)	
D43.	Other important information about ART provision at this site:
D44.	End time this section.

PLEASE PROCEED TO NEXT SECTION (SECTION E)

Section E**: Laboratory Services**

Coding purposes

1e.	Country site	
2e.	Name of health facility	
3e.	District	

4e.	Informed consent obtained from interviewee(s)	[1] Yes [2] No
	**If no, please obtain informed consent before proceeding, and start again.**	

5e.	Date of interview	
6e.	Start time of interview	

7e.	Name of interviewee 1	
8e.	Professional qualification of interviewee 1	[1] Specialist physician [2] Generalist physician<br>[3] Clinical officer [4] Medical assistant<br>[5] Trained midwife [6] Professional nurse<br>[7] Enrolled nursing assistant [8] Enrolled nurse<br>[9] Counsellor [10] Nurse counsellor<br>[11] Community health worker/health surveillance asst.<br>[12] Other *(specify)*

9e.	Name of interviewee 2	☐ None
10e.	Professional qualification of interviewee 2	[1] Specialist physician [2] Generalist physician<br>[3] Clinical officer [4] Medical assistant<br>[5] Trained midwife [6] Professional nurse<br>[7] Enrolled nursing assistant [8] Enrolled nurse<br>[9] Counsellor [10] Nurse counsellor<br>[11] Community health worker/health surveillance asst.<br>[12] Other *(specify)*

11e.	Name(s) of interviewer(s)	

PROCEED TO NEXT PAGE.

Section E**: Laboratory Services**

Coding purposes

	Does this facility provide or refer for any of the following laboratory services?	
E1.	Haemoglobin	[1] Yes, on site [2] Yes, refer to another site [3] Not available<br>If [2] Refer, then list referral site:
E2.	Total white cell count (WCC)	[1] Yes, on site [2] Yes, refer to another site [3] Not available<br>If [2] Refer, then list referral site:
E3.	Differential WCC	[1] Yes, on site [2] Yes, refer to another site [3] Not available<br>If [2] Refer, then list referral site:
E4.	Liver enzymes	[1] Yes, on site [2] Yes, refer to another site [3] Not available<br>If [2] Refer, then list referral site:
E5.	Urea and creatnine	[1] Yes, on site [2] Yes, refer to another site [3] Not available<br>If [2] Refer, then list referral site:
E6.	Electrolytes	[1] Yes, on site [2] Yes, refer to another site [3] Not available<br>If [2] Refer, then list referral site:
E7.	Glucose	[1] Yes, on site [2] Yes, refer to another site [3] Not available<br>If [2] Refer, then list referral site:
E8.	Lipids	[1] Yes, on site [2] Yes, refer to another site [3] Not available<br>If [2] Refer, then list referral site:
E9.	Amylase	[1] Yes, on site [2] Yes, refer to another site [3] Not available<br>If [2] Refer, then list referral site:
E10.	Lactate	[1] Yes, on site [2] Yes, refer to another site [3] Not available<br>If [2] Refer, then list referral site:
E11.	Pregnancy test (B-HCG)	[1] Yes, on site [2] Yes, refer to another site [3] Not available<br>If [2] Refer, then list referral site:
E12.	Rapid test for malaria	[1] Yes, on site [2] Yes, refer to another site [3] Not available<br>If [2] Refer, then list referral site:
E13.	Blood microscopy for malaria	[1] Yes, on site [2] Yes, refer to another site [3] Not available<br>If [2] Refer, then list referral site:
E14.	Cryptococcal antigen titre (CrAg)	[1] Yes, on site [2] Yes, refer to another site [3] Not available<br>If [2] Refer, then list referral site:
E15.	CSF microscopy	[1] Yes, on site [2] Yes, refer to another site [3] Not available<br>If [2] Refer, then list referral site:
E16.	CSF microscopy with India Ink	[1] Yes, on site [2] Yes, refer to another site [3] Not available<br>If [2] Refer, then list referral site:
E17.	Blood culture	[1] Yes, on site [2] Yes, refer to another site [3] Not available<br>If [2] Refer, then list referral site:

Coding purposes

E18.	Sputum microscopy for TB	[1] Yes, on site [2] Yes, refer to another site [3] Not available<br>If [2] Refer, then list referral site:
E19.	Sputum culture for TB	[1] Yes, on site [2] Yes, refer to another site [3] Not available<br>If [2] Refer, then list referral site:
E20.	TB drug sensitivity testing	[1] Yes, on site [2] Yes, refer to another site [3] Not available<br>If [2] Refer, then list referral site:
E21.	Chest X-ray	[1] Yes, on site [2] Yes, refer to another site [3] Not available<br>If [2] Refer, then list referral site:
E22.	CD4 count	[1] Yes, on site [2] Yes, refer to another site [3] Not available<br>If [2] Refer, then list referral site:
E23.	Viral load for HIV	[1] Yes, on site [2] Yes, refer to another site [3] Not available<br>If [2] Refer, then list referral site:
E24.	HIV drug resistance testing	[1] Yes, on site [2] Yes, refer to another site [3] Not available<br>If [2] Refer, then list referral site:
E25.	Rapid HIV test	[1] Yes, on site [2] Yes, refer to another site [3] Not available<br>If [2] Refer, then list referral site:
E26.	Other test *(specify):*	[1] Yes, on site [2] Yes, refer to another site<br>If [2] Refer, then list referral site:
E27.	Other test *(specify):*	[1] Yes, on site [2] Yes, refer to another site<br>If [2] Refer, then list referral site:
E28.	Other test *(specify):*	[1] Yes, on site [2] Yes, refer to another site<br>If [2] Refer, then list referral site:

E29.	Does this facility charge for laboratory tests?<br>*If yes, describe the costs and amounts.*	[1] Yes [2] No

E30.	What is the **main** funder for laboratory services at your facility?	
		[1] Government [2] NGO<br>[3] Private institution [4] Research institution/University<br>[5] Faith-based provider [6] Don't know<br>[7] Other ____________________

E31.	How many CD4 tests were conducted at this facility in the months listed below?	
	# CD4 tests SEPTEMBER 2011	☐ None
	# CD4 tests AUGUST 2011	☐ None
	# CD4 tests JULY 2011	☐ None

Coding purposes

E32.	Is there a transport system for laboratory specimens at this facility? *If yes, describe the transport system.*	[1] Yes [2] No
E33.	Is there any point-of-care laboratory testing available at your site? *If yes, describe the point-of-care tests.*	[1] Yes [2] No
E34.	Is there any other important information about laboratory services at this facility?	
E35.	End time of this section	

END OF SURVEY.
