# Supplementary material for: The Lablite project: A cross-sectional mapping survey of decentralized HIV service provision in Malawi, Uganda and Zimbabwe
Source: BMC Health Serv Res. 2014 Aug 19;14:352. doi: 10.1186/1472-6963-14-352 (PMC4148932; doi:10.1186/1472-6963-14-352)
Supplement: Supplementary file 2 — Additional file 2: Chan_Supplementarytables. (DOC 142 KB) [file 12913_2014_3455_MOESM2_ESM.doc]

Additional file 2: Table S1 ART regimens

A1(a) Adult ART regimens used in health facilities providing ART on-site

|  |  | Malawi | | | Uganda | | | Zimbabwe | |
| --- | --- | --- | --- | --- | --- | --- | --- | --- | --- |
|  |  | Primary | Secondary | Tertiary | Primary | Secondary | Tertiary | Primary | Secondary |
|  |  | (n=9) | (n=3^1^) | (n=1) | (n=6) | (n=16) | (n=2) | (n=15) | (n=6) |
| First-line regimens | | | | | | | | | |
|  | TDF/3TC/NVP | 1 | 0 | 0 | 1 | 10 | 2 | 6 | 2 |
|  | TDF/3TC/EFV | 3 | 1 | 1 | 3 | 10 | 2 | 3 | 1 |
|  | TDF/3TC/NNRTI^2^ | - | - | - | 1 | - | - | 2 | 2 |
|  | D4T/3TC/NVP | 9 | 2 | 1 | 0 | 2 | 0 | 8 | 3 |
|  | D4T/3TC/EFV | 3 | 0 | 0 | 0 | 1 | 0 | 0 | 0 |
|  | ZDV/3TC/NVP | 4 | 1 | 0 | 6 | 15 | 2 | 2 | 1 |
|  | ZDV/3TC/EFV | 1 | 1 | 0 | 6 | 8 | 2 | 1 | 0 |
|  | ZDV/3TC/NNRTI^2^ | 1 | - | - | - | - | - | - | 3 |
|  | ABC/3TC/NVP | 0 | 0 | 0 | 0 | 1 | 0 | 0 | 0 |
|  | ABC/3TC/EFV | 0 | 0 | 0 | 0 | 2 | 0 | 0 | 0 |
|  | EFV-based^3^ | 1 | - | - | - | - | - | - | - |
|  | Kaletra-based | 0 | 0 | 0 | 0 | 1 | 0 | 0 | 0 |
| Second-line availability | | | | | | | | | |
|  | Available | 1 | 2 | 1 | 4 | 13 | 2 | 0 | 4 |
|  | Refer | 8 | 1 | 0 | 2 | 2 | 0 | 15 | 2 |
|  | No second line | 0 | 0 | 0 | 0 | 1^5^ | 0 | 0 | 0 |
| Second-line regimens^4^ | | | | | | | | | |
|  | Kaletra-based |  |  |  | 4 | 13 | 2 |  | 3 |
|  | Atazanavir-based |  |  |  | 0 | 0 | 1 |  | 0 |

^1^No information on first-line regimens for one facility.

^2^Not specified whether EFV or NVP (not included here if information provided).

^3^No further information.

4Information only available for facilities with available second line. No information on second-line regimens for any facilities in Malawi, for one secondary care facility in Zimbabwe.

^5^Uses alternative first-line.

S1(b) Paediatric ART regimens used in health facilities providing ART on-site

|  |  | Malawi |  |  | Uganda |  |  | Zimbabwe |  |
| --- | --- | --- | --- | --- | --- | --- | --- | --- | --- |
|  |  | Primary | Secondary | Tertiary | Primary | Secondary | Tertiary | Primary | Secondary |
|  |  | (n=9) | (n=3) | (n=1) | (n=6) | (n=16) | (n=2) | (n=5^1^) | (n=5^2^) |
| First-line regimens | | | | | | | | | |
|  | TDF/3TC/NVP | 0 | 0 | 0 | 1 | 2 | 0 | 0 | 0 |
|  | TDF/3TC/EFV | 0 | 0 | 0 | 0 | 1 | 0 | 0 | 0 |
|  | D4T/3TC/NVP^*^ | 3 | 1 | 0 | 2 | 8 | 0 | 3 | 0 |
|  | D4T/3TC/EFV | 1 | 0 | 0 | 1 | 1 | 0 | 0 | 0 |
|  | ZDV/3TC/NVP** | 8 | 2 | 1 | 5 | 14 | 2 | 2 | 5 |
|  | ZDV/3TC/EFV | 2 | 1 | 0 | 1 | 6 | 2 | 0 | 0 |
|  | ABC/3TC/NVP | 0 | 0 | 0 | 0 | 4 | 1 | 0 | 0 |
|  | ABC/3TC/EFV | 0 | 0 | 0 | 0 | 1 | 1 | 0 | 0 |
|  | NVP syrup | 0 | 0 | 0 | 0 | 1 | 0 | 0 | 0 |
|  | Kaletra-based^#^ | 0 | 0 | 0 | 1 | 0 | 1 | 0 | 1 |
| Second-line availability | | | | | | | | | |
|  | Available | 3^3^ | 1^4^ | 1^4^ | 4 | 12^5^ | 2 |  | 1 |
|  | Refer | 6 | 2 | 0 | 2 | 2 | 0 |  | 4 |
|  | No second line | 0 | 0 | 0 | 0 | 1 | 0 |  | 0 |
| Second-line regimens | | | | | | | | | |
|  | Kaletra-based^##^ | 1 |  |  | 4 | 9 | 1 |  |  |
|  | D4T/3TC/NVP | 0 |  |  | 0 | 1 | 0 |  |  |
|  | ABC/3TC/ZDV | 0 |  |  | 0 | 0 | 1 |  |  |
|  | ABC/3TC/NVP | 0 |  |  | 1 | 1 | 0 |  |  |
|  | ABC/3TC/EFV | 0 |  |  | 1 | 0 | 0 |  |  |
|  | ABC.3TC/NNRTI^###^ | 0 |  |  | 0 | 1 | 0 |  |  |

^1^In Zimbabwe 3 primary care facilities did not do paediatric ART initiation on-site; only 5 of the remainder (where provision was by outreach teams) provided information. No information available on second-line regimens.

^2^In Zimbabwe 1 secondary care facility provided no information on paediatric regimens.

^3^No information on second-line regimens for 2/3 facilities.

^4^No information on second-line regimens.

^5^One facility had no information on availability of second line.

*Paediatric formulation of D4T/3TC/NVP specified for 1/3, 0/1, 0, 0/2, 2/8, 0, 3/3 and 0 facilities respectively

**Paediatric formulation of ZDV/3TC/NVP specified for 5/8, 1/2, 1/1, 1/5, 2/14, 0/2, 1/2, 2/5 facilities respectively

^#^Of first-line kaletra based regimens, kaletra-syrup specified for primary care facility in Uganda

^##^Of second-line kaletra based regimens 1/1 in Malawi included ABC, 1/4, 2/9, 0/1 included ABC in Uganda and an additional 1/9 secondary care facilities in Uganda specified kaletra-syrup

^###^ Not specified whether EFV or NVP (not included here if information provided).

S1(c) PMTCT used in health facilities providing PMTCT on-site

|  |  | Malawi |  |  | Uganda |  |  | Zimbabwe |  |
| --- | --- | --- | --- | --- | --- | --- | --- | --- | --- |
|  |  | Primary | Secondary | Tertiary | Primary | Secondary | Tertiary | Primary | Secondary |
|  |  | (n=15) | (n=3) | (n=1) | (n=21) | (n=16) | (n=2) | (n=16) | (n=3^1^) |
| Mothers | | | | | | | | | |
|  | Option A | 4 | 1 | 0 | 10 | 7 | 1 | 5 | 1 |
|  | Modified Option A^2^ | 2 | 0 | 0 | 8 | 8 | 1 | 11 | 1 |
|  | TDF/3TC/EFV | 9 | 2 | 1 | 0 | 0 | 0 | 0 | 1 |
|  | TDF/3TC/NVP | 0 | 1 | 0 | 0 | 0 | 0 | 0 | 0 |
|  | ZDV/3TC/NVP | 0 | 0 | 0 | 1 | 1 | 0 | 0 | 0 |
|  | NVP alone | 1 | 0 | 0 | 2 | 0 | 0 | 0 | 0 |
| Infants | | | | | | | | | |
|  | NVP | 9 | 2 | 1 | 21 | 12 | 2 | 15 | 3 |
|  | ZDV | 1 | 0 | 0 | 0 | 2 | 0 | 0 | 0 |
|  | NVP+ZDV | 2 | 0 | 0 | 0 | 0 | 0 | 0 | 0 |
|  | ZDV/3TC/NVP | 1 | 0 | 0 | 0 | 0 | 0 | 0 | 0 |
|  | Paediatric D4T/3TC/NVP | 0 | 0 | 0 | 0 | 0 | 0 | 1 | 0 |
|  | Refers children | 1 | 0 | 0 | 0 | 1 | 0 | 0 | 0 |

Information on infant regimen missing for one primary care facility in Malawi, one secondary care facility in Malawi and one secondary care facility in Uganda.

^1^No information on regimens for 3/6 secondary care facilities in Zimbabwe.

^2^These sites provided Option A but did not use all of the components.

**Table S2 CD4 tests performed per month in health facilities providing ART on-site with data available**

|  |  | Malawi |  |  | Uganda |  |  | Zimbabwe |  |
| --- | --- | --- | --- | --- | --- | --- | --- | --- | --- |
|  |  | Primary | Secondary | Tertiary | Primary | Secondary | Tertiary | Primary | Secondary |
| All facilities | | (n=2) | (n=1) | (n=1) | (n=4) | (n=14^$^) | (n=1) | (n=7) | (n=6) |
|  | CD4s per month | 84 (77-91) | 57 | 831 | 40 (27-141) | 82 (19-152) | 388 | 10 (6-30) | 171 (12-312) |
|  | Adults* on ART | 201; missing | 9,600 | 17,453 | 189 (74-705) | 576 (336-874) | 2,412 | 263 (129-1,845) | 2,800 (1,976-3,726) |
|  | Adults initiating ART per month | 46 (40-52) | 478 | 597 | 11 (7-22) | 27 (12-34) | 35 | 13 (6-20) | 50 (40-100) |
| Facilities where adults initiating ART per month ≥5% total adults* on ART | | (n=1) | (n=0) | (n=0) | (n=2) | (n=5^$$^) | (n=0) | (n=3#) | (n=1) |
|  | CD4s per month | 91 |  |  | 27 (25-29) | 102 (63-127) |  | 10 (7-17) | 312 |
|  | Adults* on ART | 201 |  |  | 74 (56-92) | 302 (103-662) |  | 129 (111-359) | 2,385 |
|  | Adults initiating ART per month | 40 |  |  | 7 (6-8) | 32 (30-34) |  | 15 (13-20) | 184 |
| Facilities where adults initiating ART per month <5% total adults* on ART | | (n=0) | (n=1) | (n=1) | (n=2) | (n=9^$$$^) | (n=1) | (n=4) | (n=5) |
|  | CD4s per month |  | 57 | 831 | 141 (50-233) | 41 (18-152) | 388 | 18 (6-81) | 152 (12-189) |
|  | Adults* on ART |  | 9,600 | 17,453 | 705 (286-1,123) | 825 (523-905) | 2,412 | 1,054 (233-2,197) | 3,214 (1,976-3,726) |
|  | Adults initiating ART per month |  | 478 | 597 | 22 (14-29) | 14 (12-33) | 35 | 7 (3-38) | 50 (40-50) |
|  | Estimated frequency of CD4s in adults* on ART** |  |  |  |  |  |  |  |  |
|  | At least 6-monthly |  | 0 | 0 | 2 | 2 | 0 | 0 | 0 |
|  | Every 6 months to 1 year |  | 0 | 0 | 0 | 2 | 1 | 0 | 1 |
|  | Every 1 to 2 years |  | 0 | 1 | 0 | 2 | 0 | 1 | 1 |
|  | Less than once every 2 years |  | 1 | 0 | 0 | 3 | 0 | 3 | 3 |

Values are median (IQR) or n.

Sample collection and testing was on-site at all facilities in Malawi, tertiary care facilities in Uganda and secondary care facilities in Zimbabwe. Samples were taken on-site and sent to a referral laboratory at all primary care facilities in Uganda and Zimbabwe

^$^Sample collection and testing on-site (n=7), samples taken on-site and sent to a referral laboratory (n=6), patients sent to a referral laboratory (n=1)

^$$^Sample collection and testing on-site (n=3), samples taken on-site and sent to a referral laboratory (n=2)

^$$$^Sample collection and testing on-site (n=4), samples taken on-site and sent to a referral laboratory (n=4), patients sent to a referral laboratory (n=1)

*Current adult patients at facility (except for Malawi where numbers are all ART patients)

** Estimated assuming constant number of patients on ART and constant rate of CD4-testing (includes all CD4s although some will be for initiation or in children). Of these facilities 0/1, 0/1 facilities in Malawi, 2/2, 6/9, 1/1 facilities in Uganda and 2/4, 2/5 facilities in Zimbabwe reported CD4-monitoring in adults was at least 6-monthly (across non-empty cells).

Note: all facilities and the final row of the table (Estimated frequency of CD4s in adults on ART in facilities where adults initiating ART per month <5% of total adults on ART) are shown in table 6.
